# Hemophilia A ameliorated in mice by CRISPR-based *in vivo* genome editing of human Factor VIII

**DOI:** 10.1038/s41598-019-53198-y

**Published:** 2019-11-14

**Authors:** Hainan Chen, Mi Shi, Avital Gilam, Qi Zheng, Yin Zhang, Ivka Afrikanova, Jinling Li, Zoya Gluzman, Ruhong Jiang, Ling-Jie Kong, Ruby Yanru Chen-Tsai

**Affiliations:** Applied Stemcell, Inc., 521 Cottonwood Drive, Milpitas, CA 95035 USA

**Keywords:** Genetics, Genomics

## Abstract

Hemophilia A is a monogenic disease with a blood clotting factor VIII (FVIII) deficiency caused by mutation in the factor VIII (*F8)* gene. Current and emerging treatments such as FVIII protein injection and gene therapies via AAV-delivered *F8* transgene in an episome are costly and nonpermanent. Here, we describe a CRISPR/Cas9-based *in vivo* genome editing method, combined with non-homologous end joining, enabling permanent chromosomal integration of a modified human B domain deleted-F8 (*BDD-F8)* at the albumin (*Alb*) locus in liver cells. To test the approach in mice, C57BL/6 mice received tail vein injections of two vectors, AAV8-SaCas9-gRNA, targeting *Alb* intron 13, and AAV8-*BDD-F8*. This resulted in *BDD-F8* insertion at the *Alb* locus and FVIII protein expression in the liver of vector-, but not vehicle-, treated mice. Using this approach in hemophilic mice, *BDD-F8* was expressed in liver cells as functional human FVIII, leading to increased plasma levels of FVIII and restoration of blood clotting properties in a dose-dependent manor for at least 7 months, with no detectable liver toxicity or meaningful off-target effects. Based on these findings, our *BDD-F8* genome editing approach may offer an efficacious, long-term and safe treatment for patients with hemophilia A.

## Introduction

Hemophilia is a group of inherited hemorrhagic disorders resulting from defects or deficiency of critical coagulating factors^1,2^. Without those functional factors, coagulating cascades, including thrombin generation and fibrin clot formation are severely compromised. Hemophilia patients suffer from spontaneous bleeding episodes, most often in joints, internal organs, and intracranially, as well as excessive bleeding associated with surgery or trauma^2^.

About 70–80% of hemophilia cases are hemophilia A, which is an X-linked condition, primarily affecting males. The mean prevalence of hemophilia A in high-income countries is 12.8 ± 6.0 per 100,000 males and is about 6.6 ± 4.8, in the rest of the world^3^. Hemophilia A is caused by various mutations in the *F8* gene, resulting in a deficiency of functional factor VIII protein (FVIII). FVIII is mainly expressed in human liver sinusoidal endothelial cells (LSECs), not hepatocytes, in human liver^4^. FVIII participates in blood coagulation as a cofactor for factor IXa that, in the presence of Ca^2+^ and phospholipids, converts factor X to its activated form Xa^5–8^. Clinical severity of hemophilia A may be mild (5%–50% of normal FVIII activity), moderate (1%–5% of normal FVIII activity), or severe (<1% of normal FVIII activity). In moderate cases, patients bleed after injuries, and also suffer spontaneous bleeding episodes without obvious cause. Patients with severe hemophilia have recurrent spontaneous bleeding episodes, in addition to their bleeding complications from normal injuries^9,10^, that can be life-threatening and require immediate medical attention.

Hemophilia A is currently treated by replacement therapies, parenteral delivery of plasma-derived or recombinant FVIII concentrates. Patients with severe disease receive prophylactic infusions of FVIII concentrate, either 3 times per week or every other day, to provide Factor VIII clotting activity above 1% normal levels. This eliminates nearly all spontaneous bleeding and prevents chronic joint disease. This replacement therapy, however, is limited by frequent intravenous infusion and potential immune response to the infused concentrate, resulting in inhibitory antibodies that leave patients susceptible to increased morbidity and disability^11^. Currently, relatively longer half-lives of recombinant products^12,13^ and complex remedy using a combination of multiple activated blood clotting factors^10,11^ have been developed to improve the outcomes of replacement therapies.

Adeno-associated virus (AAV) vector-based gene therapy is an emerging novel strategy for treating hemophilia. AAV vectors show promise for both safety and delivery efficiency, for example, to deliver genes into the human liver^14^. A first-in-human clinical trial of an AAV5 vector for treating acute intermittent porphyria (AIP) concluded that the therapy was safe^15^. AAV2^16^ and AAV8^17,18^ vectors were also used for treating hemophilia B already in clinical trials. But for hemophilia A gene therapy, full-length *F8* cDNA (~7 kb) does not fit within recombinant AAV (rAAV) vector since its DNA packaging size is limited to ≤5 kb. Even though larger transgenes have been packaged at >105% of WT size, the resulting rAAV was heterogeneous in genome size and infectivity was decreased by several logs^19^. Intriguingly, the B-domain of factor VIII, encoded by one ~3 kb exon, was reported as uncritical for FVIII pro-coagulation activity^20^. A minimum promoter driven B-domain-deleted FVIII (BDDFVIII, 4.4 kb) was packaged in AAV, producing modest FVIII expression^21^. A more recent strategy introducing codon-optimized human BDDFVIII was significantly effective in preclinical animal models^22^. BioMarin described a phase 1/2 clinical trial in patients with severe hemophilia A using an AAV5/codon-optimized B-domain-deleted FVIII, that had been modified for improved human FVIII secretion^23^.

Limitations of an AAV-based gene therapy strategy, however, still remain. In particular, therapeutic gene expression delivered by AAV is temporary, because the viral genome containing the gene remains in the cell as an episome. There is the potential for the transgene to be diluted out over time if the vector infected cells replicate at a high rate (e.g. the hepatocytes) and thus vector based gene therapy may not be a good choice to treat pediatric patients. Also, repeat administration is generally prohibited because of humoral immune responses against AAV capsid proteins, developing after the first administration^24^. To overcome such limitations, genome editing to correct mutated genes has been investigated^25^, potentially providing a life-long solution for hemophilia patients. Targeted genome editing methods using programmable nucleases, including zinc-finger nuclease (ZFN), transcription activator-like effector nuclease (TALEN), and CRISPR/Cas9 systems^26^ have been described. These procedures all generate double-strand breaks (DSBs) at specific genomic loci. In accordance with cell cycle stage and presence or absence of a donor template, typical approaches for repairing nuclease-induced DSBs are error-prone non-homologous end joining (NHEJ) at a high repair efficiency or error-free but inefficient homology-directed repair (HDR)^24^.

In the current study, we developed a genome editing therapy utilizing two rAAV vectors, encoding *Staphylococcus aureus* Cas 9-guide RNA (SaCas9-gRNA) and a codon-optimized human B-domain-deleted human FVIII (*BDD-F8)*, respectively. This strategy aimed to use CRISPR/Cas9 genome targeting/cutting capability, combined with NHEJ DNA repair, to integrate human *BDD-F8* in a site-specific manner at the liver-specific albumin (*Alb)* locus, leading to FVIII production in the liver. Here, we evaluated the approach in mice, using mouse-specific viral vectors to achieve integration of *BDD-F8* into the mouse *Alb* locus. In FVIII knockout (F8KO) mice, a model for hemophilia A, this treatment ameliorated the disease phenotype, promoting elevated FVIII protein and activity levels in the liver for up to 7 months, without discernable liver toxicity.

## Methods

All protocols involving use of animals were approved by the Institutional Animal Care and Use Committee (IACUC) of the NASA Ames Research Center (Mountain View, CA, USA) under the protocol No. APP-16-001-Y2. All experiments were performed in accordance with relevant guidelines and regulations.

### Plasmid construction and AAV8 vector production

A pCDNA4/Full-length F8 plasmid containing full-length human WT *F8* cDNA, driven by a cytomegalovirus (CMV) promoter, was obtained from Addgene (catalogue #41036, Cambridge, MA, USA). A codon-optimized *BDD-F8* variant, containing a modified SQ/N6 sequence and an amino acid substitution at R1645H, was synthesized by GeneScript (Piscataway, NJ, USA) and sub-cloned into a pCDNA4 backbone, using the flanking *BsiWI* and *AgeI* restriction sites, with an In-Fusion cloning kit (Takra Bio, Mountain View, CA, USA), creating a pCDNA4/BDD-F8 plasmid (sequence available upon request). To target the specific intron 13 region of the mouse *Alb* locus, 5 single guide RNA (sgRNA) sequences were selected by the online DESKGEN CRISPR tool, and were each cloned into a pX602-AAV-TBG:SaCas9;U6:BsaI-sgRNA plasmid (pX602-AAV, #61593 from Addgene) at the *BsaI* restriction site. To transfect human embryonic kidney 293 (HEK-293) cells, the TBG promoter in those plasmids was used to replace a CBh promoter. The donor plasmid, containing *BDD-F8* cDNA, which was preceded by P2A, mouse *Alb* exon 14 coding, and intron 13 partial splicing sequences, and was further flanked by two gRNA-PAM sequences on the 5′- and 3′-ends, was engineered by aligning three gBlock gene fragments (synthesized by Integrated DNA Technologies (IDT, Skokie, IL, USA)) into the pX602-AAV plasmid backbone at the *XbaI* and *NotI* restriction sites. Both AAV plasmids, pX602-AAV-*BDD-F8* and pX602-*SaCas9-sgRNA*, containing two AAV inverted terminal repeat (ITR) sequences, were submitted to Vigene Biosciences (Rockville, MD, USA) for AAV serotype 8 vector packaging using a triple transfection protocol produced in HEK-293 cells. The titers of the AAV vectors were 2.2 × 10^13^ vector genomes (vg) per ml for *AAV-SaCas9-sgRNA* and 1.15 × 10^13^ vg/ml for *AAV-BDD-F8*, measured by droplet digital polymerase chain reaction (ddPCR) developed in house (see below).

### AAV8 BDD-F8 and AAV8 SaCas9 viral genome titering by Droplet Digital PCR (ddPCR)

The general protocol of ddPCR is described in Gobert *et al*.^27^. Virus samples were serially diluted in DDW and were tested in replicates. Each ddPCR reaction included diluted virus sample, ddPCR Supermix for Probes - No dUTP (Bio-Rad), 900 nM of each primer (IDT) and 250 nM probe (IDT). No-template ddPCR reaction wells, in which nuclease-free water were added instead of virus sample, were used as negative control. Assays for saCas9 and BDD-F8 abundance were designed with target FAM-labeled hydrolysis probes (Suppl. Table 1).

Twenty microliters of the ddPCR reaction were loaded on 8-well cartridge to generate ~20,000 nanoliter-sized droplets, using QX200 droplet generator apparatus (Bio-Rad). The 40ul emulsion was transferred to a 96-well semi-skirted PCR plate (Bio-Rad) and was sealed with a pierceable foil heat seal in the PX1™ PCR Plate Sealer (Bio-Rad). PCR amplification was done on a Bio-Rad C1000 Touch Thermal Cycler. The following PCR program was run: denaturation at 95 °C for 10 min, 40 cycles of 94 °C for 30 s and 60 °C for 1 min, followed by 10 min denaturation at 98 °C and 4 °C hold until droplets were read. The sample plate was transferred to the Bio-Rad QX200 Droplet Reader and the fluorescence data was collected and analyzed using the QuantaSoft analysis Pro software (Bio-Rad). Droplets were considered positive when fluorescence amplitude was above the background threshold. ddPCR well acceptance criteria included >14,000 droplets count per well and <5 positive droplets in the No-template negative control well. Poisson distribution curve was used to determine the viral genome concentration of BDD-F8 and SaCas9 viruses from the ratio of positive/total droplets, and the virus genome titer was calculated based on the sample dilution(s).

### Cell culture and gene transfer

HEK-293 (ATCC, Manassas, VA, USA), mouse embryonic fibroblast (MEF, Applied StemCell Cat# AS-1116), and mouse Hepa1-6 (ATCC) cell lines were routinely maintained in complete Dulbecco’s modified Eagle’s Medium (DMEM) supplemented with 10% fetal bovine serum (FBS), 2 mM GlutaMAX, 100 U/ml penicillin, and 100 μg/ml streptomycin at 37 °C in an incubator equilibrated with 95% air, 5% CO_2_. Cells were seeded into 24-well plates at a density of 200,000 cells per well, and transfected, without or with plasmids, as described in the text (2 to 4 µg per well) using a Neon electroporation transfection system (ThermoFisher Scientific, Waltham, MA, USA). Following transfection, cells were cultured, harvested, and subjected to the various analyses.

### Human full-length FVIII and BDD-FVIII protein expression and coagulation activity in HEK-293 cells

After transfection, HEK-293 cells were cultured in complete medium for 2 days. Then, the medium was exchanged for 500 µl fresh DMEM medium supplemented with 0.5% FBS only, and the cells were cultured for 2 additional days. Culture supernatants were harvested, filtered, and stored at −80 °C until assayed for FVIII protein and activity. After medium removal, the HEK-293 cells were washed twice with phosphate buffered saline (PBS) and assayed for human FVIII mRNA and protein, using real-time RT-PCR and immunofluorescence, respectively.

The amount of FVIII protein released by HEK-293 cells was measured in the harvested culture medium using a sandwich ELISA developed in-house. Briefly, the assay was conducted on a 96-well microplate pre-coated with a specific anti-human mouse FVIII antibody (GMA-8024, Green Mountain Antibodies, Burlington, VT, USA). A standard curve was prepared in DMEM culture medium containing 0.5% FBS by 2-fold serially diluting normal pooled citrated human plasma (cat # FRNCP01, Affinity Biologicals, Ontario, Canada). The FVIII concentration in the pooled human plasma was 1 IU/ml (100% of normal human FVIII). Aliquots (50 µl) of supernatant samples or human plasma standards were added to wells, in duplicate, and plates were incubated for 1 h at room temperature (RT). After washing the plates, a secondary biotinylated anti-human mouse FVIII antibody (GMA-8023, Green Mountain Antibodies) was added and plates incubated for another hour. After several washing steps, streptavidin-HRP binding, and a TMB substrate chromogenic reaction were performed. Reactions were stopped with 2 N H_2_SO_4_ and absorbances were measured at 450 nm in a BioTek (Winooski, VT, USA) microplate reader. The coagulation activity of FVIII released by the HEK-293 cells into the culture medium was measured using a COATEST SP4 FVIII CHROMOGENIX assay kit (Diapharma, West Chester, OH, USA) according to the manufacturer’s instructions, with slight modifications. Briefly, 25 µl of supernatant samples or plasma standards was added to wells, in duplicate, of a 96-well microplate, followed by addition of 50 µl phospholipid, Factor IXa, and Factor X mixture. After 5 minutes incubation, 25 µl 25 mM CaCl_2_ was added and plates were incubated for 10 minutes. Next, 50 μl chromogenic substrate S-2765 was added to each well, followed by incubation for 10 minutes. All incubation steps were at 37 °C, and the absorbance of the final reaction was determined at 405 nm using a microplate reader (BioTek) at room temperature. The FVIII activity in each sample was calculated from a standard curve prepared with normal pooled citrated human plasma (Affinity Biologicals).

For immunofluorescence staining of FVIII protein, cultured HEK-293 cells were fixed in a 2% paraformaldehyde/PBS solution and permeabilized with 0.2% Triton X-100. After washing with PBS, the cells were incubated for 1 h with blocking solution containing 5% normal goat serum and 2% BSA in PBS. The cells were then incubated with primary sheep anti-human FVIII antibody (1:100, Abcam, San Francisco, CA, USA) for 2 h at RT or overnight at 4 °C, washed with PBS, and incubated with Alexa Fluor 488 labeled secondary antibody (ThermoFisher Scientific) for 1 h at RT. The cells were then mounted with an antifade mounting medium containing DAPI (Vector Laboratories, Burlingame, CA, USA) for nuclei visualization. Images were captured and analyzed using a Leica microscope.

To measure full-length FVIII or BDD-FVIII mRNA levels in HEK-293 cells after plasmid transfection, total RNA was purified from cells using the RNeasy Mini Kit (Qiagen, Germantown, MD, USA), according to the manufacturer’s instructions. RNA (1 µg) was reverse-transcribed to cDNA using the High-Capacity cDNA Archive kit (Applied Biosystems, Foster City, CA, USA). cDNA (100 ng) was applied in each real-time PCR assay using the SYBR Green PCR Master Mix kit (Applied Biosystems) and specific primers targeting the full-length *F8* or *BDD-F8* gene, as shown in Suppl. Table 2. Gene expression was reported as a relative level, normalized to human cyclophilin mRNA content.

### PCR genotyping, sequencing, and T7EN1 cleavage assay

To verify the cutting efficiency of gRNA targeting the specific regions of the mouse *Alb* locus, genomic DNA was extracted, using QuickExtract DNA Extraction solution (Epicentre, Madison, WI, USA), from mouse MEF or Hepa 1-6 cells 4 days after transfection with the indicated SaCas9-gRNA plasmids. Targeted fragments were amplified with the GoTaq Green Master Mix PCR kit (Promega, Madison, WI, USA) using specific primers, as shown in Suppl. Table 2. PCR amplicons were either Sanger sequenced (ELIM Biopharm, Haywood, CA, USA) or examined by T7 endonuclease I (T7EN1)-based mutation detection assay, according to the manufacturer’s instructions. In brief, the PCR products were denatured and re-annealed in NEBuffer 2 (New England BioLabs, Ipswich, MA, USA), then digested with T7EN1 (NEB, #M0302L) for 30 minutes, followed by 2.5% agarose gel separation and analysis.

### Animals

Mice used in this study were maintained at the animal facility at NASA Ames Research Center (Mountain View, CA, USA). All protocols involving use of animals were approved by the Institutional Animal Care and Use Committee (IACUC) of the NASA Ames Research Center. C57BL/6 mice were from the Jackson Laboratory (Bar Harbor, ME, USA). The *F8KO* mouse was generated in-house using CRISPR/Cas9 technology. In brief, two guide RNA molecules targeting exon 4 of the mouse *F8* locus, along with SpCas9 protein, were co-injected into C57BL/6 J mouse embryos. A male founder mouse was generated with a deletion of 37 bp nucleotides in exon 4 of the *F8* gene. The founder was bred with WT C57BL/6 J female mice to establish hemizygous/heterozygous offspring. Breeding between the hemizygous/heterozygous littermates generated homozygous females and hemizygous males, which were the *F8KO* mice used in this study.

### AAV viral vector injection in mice

To deliver AAV vectors, a mixture of AAV8-SaCas9-g1 and AAV8-BDD-F8 vectors, at a ratio of 1:2.5, 1:5, or 1:20, was administered intravenously to 6- to 8-week old male C56BL/6 or *F8KO* mice via lateral tail vein injection. All doses of AAV vectors were adjusted to 100 µl with sterile PBS, pH 7.4 before injection. The control cohorts (Vehicle) were injected with 100 µl PBS. A preliminary test revealed that the 1:5 ratio of AAV8-SaCas9-g1 to AAV8-BDD-F8 vectors produced the highest knock-in efficiency, so this ratio was used for all other experiments, unless otherwise specified. After AAV injection, mice were euthanized at specific time points, as described in the figures, for blood plasma sampling *via* retro-orbital sinus bleeding and for harvesting fresh liver tissue. In some mice, a filter-paper based tail-vein bleeding test (described below) was performed to evaluate blood-clotting function. Both the AAV dose-response and time-course studies were conducted in *F8KO* mice. In the dose-response study, mice were injected with a single dose of mixed vectors of AAV8-SaCas9-g1 and AAV8-BDD-F8 (1:5 ratio) at either 2.4 × 10^10^, 1.2 × 10^11^, 6 × 10^11^, or 3 × 10^12^ vg/kg and analyzed after one month. In the time-course study, mice were injected with 6 × 10^11^ vg/kg total AAV vector mixture (1:5 ratio) and samples were collected at 0.5, 1, 2, 3, 4, and 7 months post injection, as indicated in the figures.

### Mouse liver histology, immunostaining, and DNA/RNA purification

Following tissue harvest, each mouse liver was divided into two halves. One half was fixed in 4% paraformaldehyde/PBS overnight and submitted to the Histo-Tec laboratory (Hayward, CA, USA) for standard histology processing, paraffin embedding, and sectioning. Liver histology was assessed after hemotoxylin and eosin (H&E) staining. Immunostaining of human BDD-FVIII protein was examined using a similar staining protocol as that described above for the HEK-293 cells. The other half of each liver was homogenized, and genomic liver DNA or total RNA was purified, using Qiagen DNeasy Blood and Tissue Kit or RNeasy Mini Kit, respectively, according to the manufacturer’s instructions. Liver total RNA (1 µg) was reverse-transcribed to cDNA and 100 ng cDNA was applied in each real-time PCR reaction to measure levels of mRNAs for specific genes, as indicated in the figures. 5′- and 3′-junction PCR analyses were performed on liver cDNA samples to validate site-specific integration of the *BDD-F8* transgene at the *Alb* locus. In addition, standard PCR or real-time quantitative PCR of mouse liver DNA was used to assess the infectivity of AAV viruses in mouse liver tissues. Specific primers used in these assays are shown in the Suppl. Table 2.

### Mouse plasma measurements

Whole blood was collected from the retro-orbital sinus of each mouse, under anesthesia, directly into microtubes containing trisodium citrate (0.106 mole/l, Sarstedt, North Rhine-Westphalia, Nümbrecht, Germany). After centrifugation, plasma was harvested and stored at −80 °C until assayed for human BDD-FVIII protein or biological activity, using similar ELISA or CHROMOGENIX F8 activity assays as described for the HEK-293 cells, except for differences in mouse plasma samples and human plasma standards preparation. All mouse plasma samples were diluted with equal volumes of FVIII-depleted human plasma (Cat #FRDP080125, Affinity Biologicals), whereas standards of normal human pooled plasma were diluted with equal volumes of pooled plasma from F8KO mice. The ratio of the mouse plasma to human plasma is maintained as 1:1 for all samples and standards. These mixture samples/standards were then diluted using sample buffer from the Chromogenix assay kit for FVIII:C measurement. Fifty µl aliquots of diluted plasma samples or standards were analyzed by ELISA; and 25 µl sample or standard aliquots, further diluted by the sample buffer, and measured by the CHROMOGENIX activity assay, according to the manufacturer’s instruction using a low range detection protocol. The level or activity of human BDD-FVIII in each mouse plasma sample was calculated as a percentage of values for normal human pooled plasma.

### Activated partial thromboplastin time (aPTT) assay

To measure clotting activity of mouse plasma, the aPTT assay, a microplate-based blood coagulation assay, was performed as previously described^28^. In brief, 30 µl mouse plasma was mixed with 30 µl activated partial thromboplastin time reagent (ThermoFisher Scientific) in each well of a 96-well plate, and plate was pre-incubated for 5 minutes at 37 °C, followed by addition of 30 µl 25 mM CaCl_2_ per well. Afterwards, the microplate was immediately placed on a microplate reader (BioTek) and kinetics of absorbance at 405 nm was determined by taking a reading every 10 seconds for 8 minutes during development of the coagulation reaction at room temperature. The aPTT was defined as the time at which the coagulation reaction reached its maximum velocity.

### AAV induced liver toxicity

To assess whether AAV administration caused liver toxicity, levels of mouse plasma albumin and the activities of plasma alanine transaminase (ALT) and aspartate aminotransferase (AST) were measured using kits from Abcam (Cambridge, UK; Cat #ab108791 for albumin, #ab105134 for ALT, #ab105135 for AST). All assays were performed according to the manufacturer’s instructions.

### Mouse tail vein bleeding time assay

To evaluate hemorrhage responses in F8KO mice, before and after AAV injection, a filter paper-based tail vein bleeding time assay was performed as described previously^29^, with modifications. As shown in Suppl. Fig. 3, each mouse was anesthetized in a chamber box using isoflurane, and positioned horizontally on a platform with the tail hung out of the box through a small hole. The mouse tail was transected by a surgical blade at the place where the cross-sectional diameter was about 1.8 mm, as judged by a drill gauge. Whatman filter paper (Sigma-Aldrich, St. Louis, MO, USA), divided into 8 equal sections, was applied to the edge of the forming clot every 4 minutes, without dislodging the clot. In some mice, multiple drops of blood, if they continued flowing from the cut during the 4-minute interval, were allowed to fall freely on the same spot of the filter paper. Blood collection was continued for 28 minutes and then bleeding was stopped by cauterizing the tail tip with a silver nitrate stick (ThemoFisher Scientific). After the blood drops on the paper were dried, the area of each blood drop was measured morphometrically using ImageJ software (National Institutes of Health, Bethesda, MD, USA) and the total blood volume of each sample was calculated based on the blood spot areas. The volume of blood was proportional to the area of the blood spot on the filter paper (Suppl. Fig. 4).

### CRISPR mediated on-target and off-target analyses by next generation sequencing (NGS)

Off-target sites of the saCas9/sg1 gRNA were predicted *in silico* using the COSMID online program (http://crispr.bme.gatech.edu), with the PAM motif set as NNGRRT and the search options containing up to 3 mismatches, 1 bp-deletion or insertion. At this setting, 8 potential off-target sites were identified, half in intronic regions, and the other half in an intergenic region, with no known genes present at these loci. Specific primers with barcode labeling across these off-target sites or on-target site at the *Alb* locus were designed (Suppl. Table 3). Using these primers, all sites analyzed were amplified by a Taq PCR master mix kit (Qiagene) from150 ng of input genomic mouse liver DNA, purified from F8KO mice receiving either AAV or vehicle injections. PCR products were purified with magnetic beads (Beckman Coulter Genomics, Chaska, MN, USA) followed by addition of Nextera XT Index primers using the Illumina Nextera XT index kit. The indexed libraries were further purified using magnetic beads and the library DNA concentration was quantified with a Synergy HTX BioTek microplate reader. Normalized library-prepared samples were then loaded onto an Illumina MiniSeq system for deep sequencing in paired-end mode. For each sample, at least 10,000 reads were obtained. Templates of the saCas9/sg1 target site and the potential off-target sites were used as a reference sequence for determining off-target events. Reference-guided sequence assemblies were performed using an in-house developed assembly platform.

### Indel and knock-In abundance quantification by droplet digital PCR (ddPCR) analysis

The general protocol of ddPCR is described in Gobert *et al*.^27^. Genomic DNA was extracted from homogenized liver tissues using High Pure PCR Template Preparation Kit (Roche, Indianapolis, IN, USA), according to the manufacturer’s instructions. ddPCR reactions were performed using ddPCR Supermix for Probes - No dUTP (Bio-Rad, Hercules, CA, USA), 900 nM of each primer, 250 nM probe, Hindlll restriction enzyme (NEB, 20 U) and 50–130 ng extracted genomic DNA. For negative controls, 2 µl water was added instead of genomic DNA. Assays for indel and KI abundance were designed with target FAM-labeled hydrolysis probes and a reference region HEX-labeled probe (Suppl. Table 4). Twenty microliters of the reaction mixture was loaded in each sample well of an 8-well cartridge to generate 20,000 droplets, using a droplet generator apparatus (Bio-Rad) according to the manufacturer’s instructions. The emulsion was transferred to a 96-well PCR plate (Bio-Rad) and amplified to the end-point in a C1000 Touch thermocycler (Bio-Rad) under the following conditions: denaturation at 95 °C for 10 minutes, 40 cycles of 94 °C for 30 seconds, and 57 °C/57.5 °C for 1/3 minutes (indel/KI, respectively), followed by 10 minutes denaturation at 98 °C and 12 °C until droplets were read. Droplet fluorescence was read with the QX200 droplet reader apparatus (Bio-Rad) and data acquisition and analysis were performed using the QuantaSoft Analysis Pro software (Bio-Rad). Droplets were considered positive when fluorescence was above the background threshold of the negative droplets of the same sample and of negative controls. The number of molecules of target DNA present in the 20 μl reaction mixture was determined from the ratio of positive/total droplets. Quality controls were done as described above (AAV8 BDD-F8 and AAV8 SaCas9 viral genome titering by Droplet Digital PCR).

### Statistical analysis

Data are presented as means ± standard errors (SEs). Poisson distribution and means were used to calculate the numbers of copies of knock-in target and Indel detected in ddPCR assays. Student’s t-test or one-way ANOVA was used for statistical analyses and P < 0.05 was considered statistically significant.

## Results

### Modified human BDD-F8 transgene expressed functional FVIII in HEK-293 cells

**Figure 1:**
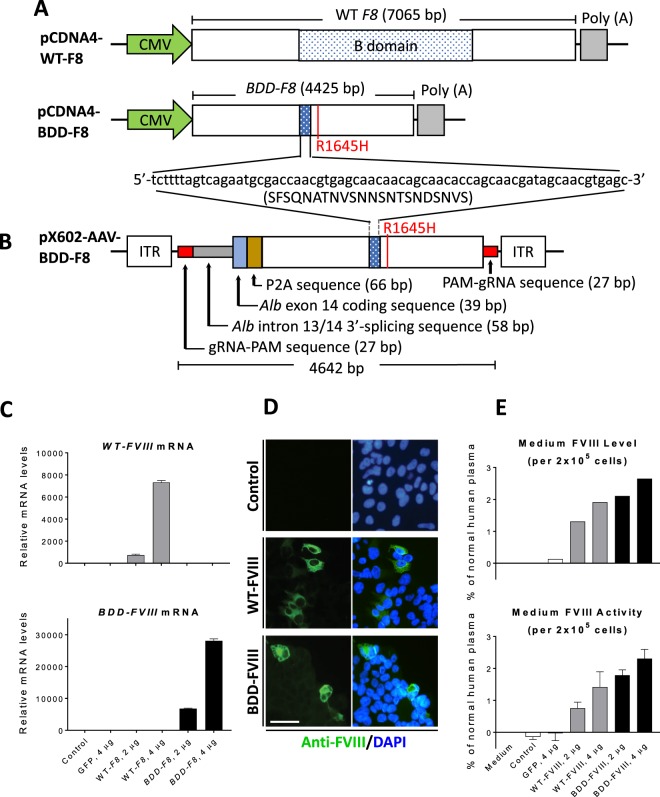
Construction and validation of transgene vectors for expression of human B domain deleted FVIII. (**A)** Schematic diagrams of pCDNA4 plasmid containing human wild-type (WT) full length *F8* cDNA (7065 bp) or modified *BDD-F8* cDNA (4425 bp), both driven by a CMV promoter. *BDD-F8* cDNA contained an amino acid substitution, R1645H (red), and a short B domain sequence (66 bp nucleotides/22 amino acid residues encoded). **(B)**The donor vector pX602-AAV-*BDD-F8* for CRISPR/Cas9-mediated insertion contains an inverted terminal repeat (ITR), gRNA-PAM sequence, 3′-splicing sequence of *Alb* intron 13, and *Alb* exon 14 codon sequence, followed by P2A, *BDD-F8* cDNA, a second gRNA-PAM sequence and another ITR. The entire *BDD-F8* cassette was 4642 bp in length. **(C–E)** FVIII expression and functional activity tests in HEK-293 cells transfected without (control) or with pCDNA4-WT-F8 or pCDNA4-BDD-F8 plasmids. At 4 days after transfection, **(C)** mRNA and **(D)** protein expression of *WT-F8* or *BDD-F8* were assessed by real-time RT-PCR and immunofluorescence, respectively. Scale bars, 10 µm. **(E)** Content and activity of FVIII or BDD-FVIII protein in the cell culture medium, released from HEK-293 cells, were measured by ELISA and Chromogenix FVIII activity assay, respectively. Data are means ± SE from 3 independent experiments, except that ELISA measurements were performed in only one of these 3 experiments.

The wild-type (WT) full-length human *F8* cDNA is >7 kb, beyond the packaging limit of recombinant AAV vectors. Variants of human FVIII with the B-domain deleted (BDD), either completely or partially, not only fit into the AAV genome, but also retained full procoagulant activity with sufficient synthesis and secretion^30^. In this study, we used a BDD-FVIII variant, containing a modified SQ/N6 sequence (Fig. 1A^31^,) but also introduced one amino acid substitution, R1645H, because this amino acid substitution was reported to yield higher FVIII activity^32^. We further codon-optimized the cDNA sequence of this BDD-FVIII variant (hereafter designated as “*BDD-F8”*), and sub-cloned it into the pCDNA4 plasmid. In HEK-293 cells, after transfection of WT *F8* (pCDNA4-WT F8) or *BDD-F8* (pCDNA4-BDD-F8) plasmids, the *BDD-F8* transgene was expressed as a different mRNA molecule than was WT *F8* (Fig. 1C). However, based on immunofluorescence, its protein expression was comparable to that of WT *F8* (Fig. 1D). The protein translated by *BDD-F8* or WT *F8* was secreted into the culture medium, as detected by an FVIII ELISA assay (Fig. 1E). Consistent with a previous report^32^, the secreted FVIII protein produced by *BDD-F8* plasmid appeared to have higher coagulating activity than that from the same amount of WT *F8* plasmid (Fig. 1E). Thus, the modified human *BDD-F8* transgene generated biologically active and secreted FVIII protein. Next, we sub-cloned the *BDD-F8* transgene into a pX602-AAV plasmid (Fig. 1B) for AAV8 virus packaging and production for *in vivo* studies.

### Validation of CRISPR gRNA targeting the mouse *Alb locus*

**Figure 2:**
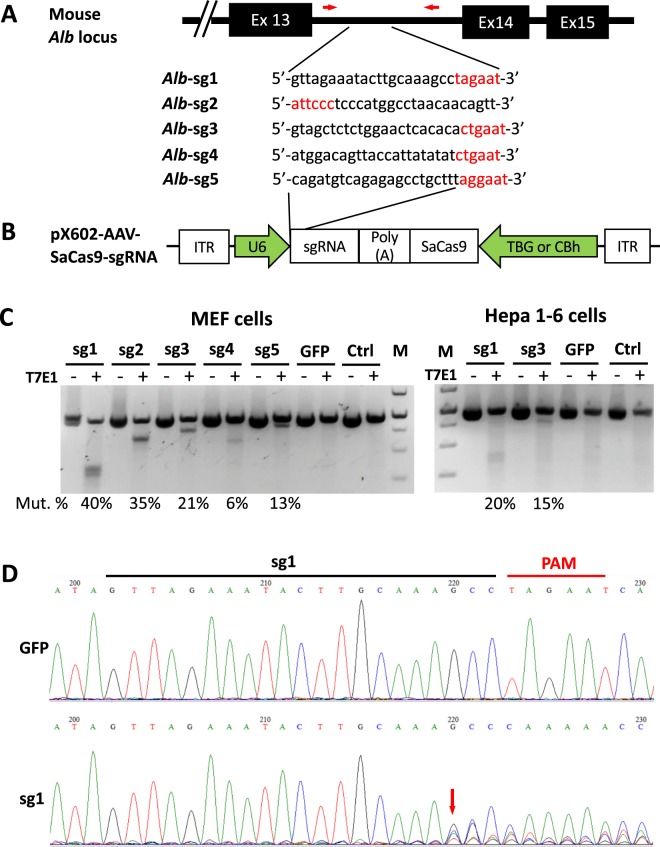
Validation of guide RNA targeting the mouse *Alb* locus. (**A**) Schematic diagrams illustrating the site of the mouse *Alb* locus for CRISPR/SaCas9-mediated genome editing. The sequences of 5 guide RNAs (gRNAs) were each cloned into a pX602-AAV-SaCas9-gRNA plasmid **(B)**. SaCas9 was driven by a TBG or CBh promoter. Mouse embryonic fibroblast cells (MEF, using pX602-AAV-CBh-SaCas9-sgRNA) or mouse Hepa 1–6 cells (using pX602-AAV-TBG-SaCas9-sgRNA) were transfected without (Ctrl) or with the plasmids, as indicated **(C,D)**. **(C)** Representative T7 endonuclease I (T7E1) mismatch cleavage assay showing CRISPR/SaCas9-mediated mutation events (Mut. %) at the target *Alb* locus. The PCR product was amplified using primers specific for the targeting region (red arrows in **A**). Image of the full-length gels are shown in Supplementary Fig. 5. **(D)** Sanger sequencing of PCR products from the MEF cells, showing the indel mutations caused by SaCas9 and gRNA1 (sg1).

**Figure 3:**
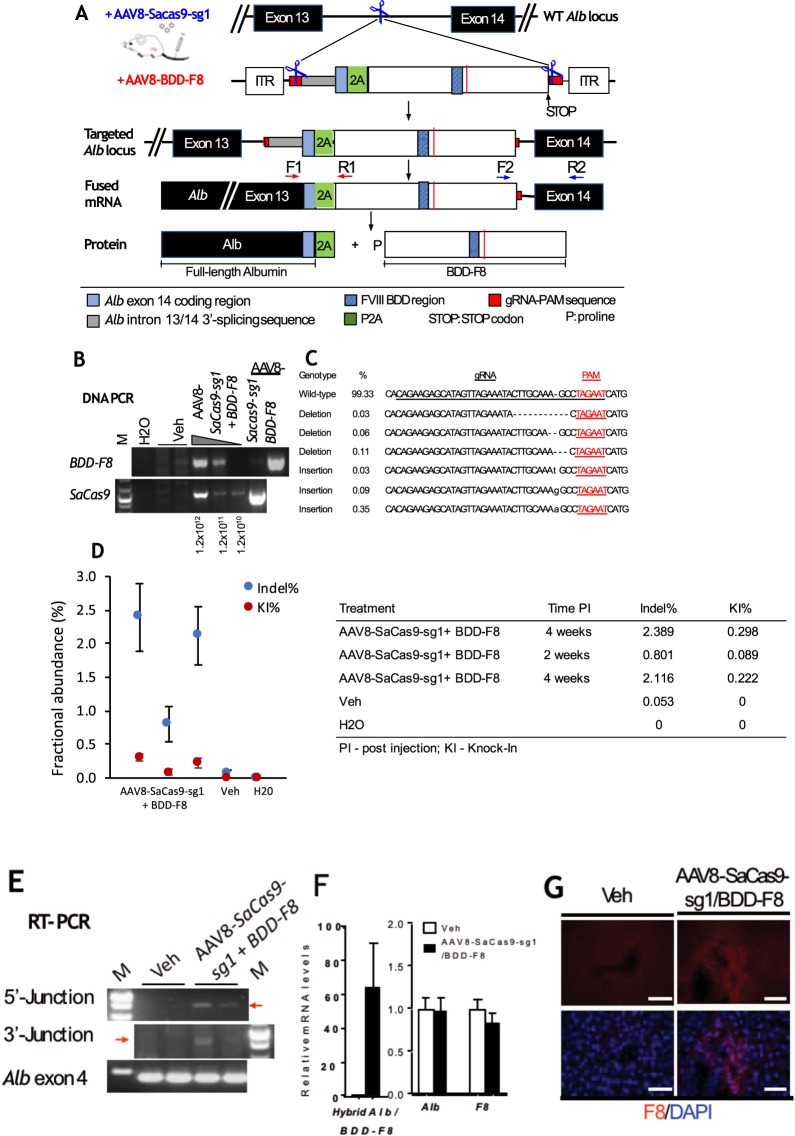
Site-specific integration and expression of human *BDD-F8* transgene into the mouse *Alb* locus. (**A**) Schematic diagrams showing the *BDD-F8* transgene knock-in strategy. Adult C57BL/6 mice received a mixture of two AAV8 vectors via tail vein injection. Vector AAV8-SaCas9-sg1 expresses SaCas9 and sg1 gRNA targeting the *Alb* intron 13 region. Vector AAV8-BDD-F8 contains the *BDD-F8* transgene cassette flanked by two sg1 gRNA recognition sequences (red boxes, also illustrated in Fig. 1A). Liver samples were collected for analyses 4 weeks after viral injection. **(B)** PCR showing viral infection in the mouse liver with vehicle only (Veh) or the two AAV8 vectors at a 1:5 ratio and total dose of 1.2 × 10^12^, 1.2 × 10^11^, or 1.2 × 10^10^ vg/kg, as indicated. M, DNA molecular weight marker. The full-length gels are shown in Supplementary Fig. 6,A. **(C)** Representative NGS data showing WT and mutated (indel) sequence patterns at the targeted *Alb* locus, and the percentage of each variant. **(D)** ddPCR analysis showing indel abundance (%) and *BDD-F8* transgene knock-in event rates (KI%) at the targeted *Alb* locus. Each column represents Poisson means ± SE. **(E)** RT-PCR analyses of mouse liver cDNA in a mouse with a total AAV dose of 1.2 × 10^12^ vg/kg. Amplicons were produced using the specific primers F1/R1 (red arrows, **A**) and F2/R2 (blue arrows, **A**) that span the 5′-junction or 3′-junction, respectively, at the insertion site. Full-length gels are shown in Supplementary Fig. 6,B–D. **(F)** Relative levels of hybrid *Alb/BDD-F8* and endogenous mouse *Alb* and *F8* mRNAs in mouse livers. Data are means ± SE from 3–4 mice per group. **(G)** Representative immunofluorescence staining images, showing sporadic expression of human BDD-FVIII protein (red) in liver sections. Scale bars, 10 µm.

To integrate human *BDD-F8* in a site-specific manner at the mouse *Alb* locus using CRISPR/Cas9, we first identified five gRNA candidates targeting the mouse *Alb* intron13 region *in silico* (Fig. 2A). The rationale to target *Alb* intron 13 instead of earlier introns or later exons or 3′-UTR sequences is to keep Albumin protein and expression intact after *BDD-F8* insertion. To that end, the last 13 amino acids of Albumin exon 14 was included in the donor (Fig. 3A) and separated from BDD-FVIII by 2 A peptide.

These gRNAs were each sub-cloned into a pX602-AAV plasmid that co-expressed *S. aureus* Cas9 (SaCas9), driven by liver-specific human thyroxine-binding globulin promoter (TBG) or a hybrid form of CMV enhancer/chicken beta-actin promoter (Cbh) (Fig. 2B). The gRNAs, denoted as *Alb*-sg1 to *Alb*-sg5, were each tested for targeting SaCas9 cleavage at the *Alb* region in either mouse embryonic fibroblast (MEF) or Hepa1-6 mouse liver cells (Fig. 2C). T7 Endonuclease I (T7EN1) mutation assays revealed that these gRNAs and SaCas9 specifically targeted and cleaved the *Alb* locus, generating mutation events at rates ranging from 6% to 40%, with *Alb*-sg1 yielding the highest mutation rate, 40% in MEF cells and 20% in Hepa 1-6 cells (Fig. 2C). Sanger sequencing results (Fig. 2D) confirmed that *Alb*-sg1 produced an incision at the expected site and created indels. Therefore, *Alb*-sg1 was chosen for CRISPR/Cas9 mediated targeting cleavage at the mouse *Alb* locus.

### *In vivo* genome editing of the human *BDD-F8* transgene into the *Alb* locus in WT mice

In mammalian cells, the NHEJ repair pathway works much more efficiently than the HDR repair pathway on double stranded DNA breaks made by nucleases such as Cas9^33^, and this was supported by our pilot experiment in HEK-293 cells (Suppl. Fig. 1). HEK-293 cells, stably expressing SpCas9, were co-transduced with two AAV2 viral vectors, a sgRNA vector targeting the *GAPDH* 3′-UTR region and a donor vector containing a GFP reporter gene led by an internal ribosome entry site (IRES) element (Suppl. Fig. 1,A). In two donor vectors, the reporter gene was flanked by either ~900 bp or ~200 bp homologous arms at each of the 5′ and 3′ ends, enabling HDR-based GFP gene integration. In the third vector, the reporter gene was flanked at each end by a gRNA/PAM sequence, which is targeted by the same sgRNA targeting the *GAPDH* 3′ UTR region, enabling Cas9 to cut out the GFP fragment and, subsequently, NHEJ-based GFP knock-in at the *GAPDH* locus (Suppl. Fig. 1,A). The AAV2 donor vector without homologous arms produced 4- to 6-fold more GFP^+^ cells than did the donor vectors with either long or short homologous arms (Suppl. Fig. 1,B,C). Thus, we chose an NHEJ-based knock-in strategy for *in vivo* insertion of the human *BDD-F8* transgene into the mouse *Alb* locus.

To achieve genome editing, two AAV8 viral vectors were constructed, one a human BDD-F8 donor vector and the other a SaCas9-gRNA vector. The donor vector (Fig. 1B, pX602-AAV-BDD-F8) has inverted terminal repeat (ITR) and gRNA targeting-PAM sequences at both 5′ and 3′ sides flanking the *BDD-F8* cassette. Cas9 cuts at the PAM sequences to release the whole donor fragment from the viral vector, allowing NHEJ-mediated gene knock-in. Preceding the *BDD-F8* cDNA sequence, there are *Alb* intron 13 3′ splicing, *Alb* exon 14 codon, and P2A sequences, such that a fused mRNA for albumin-2A ribosome-skipping peptide–human BDD-FVIII would be expressed and then processed to two separate proteins, albumin and BDD-FVIII. To accommodate AAV packaging capacity, TBG driving SaCas9, a smaller Cas9, was chosen and, along with the optimal gRNA, sg1, was cloned into the same pX602-AAV vector (Fig. 2B).

To knock in BDD-F8 at the mouse *Alb* locus, WT C57BL/6 mice received by tail vein injection a mixture of the two viral vectors, AAV8-SaCas9-sg1 and AAV8-BDD-F8, at a ratio of 1:5 (Fig. 3A). At 4 weeks after viral vector administration, both viral DNA particles were detected in mouse liver cells, by PCR analysis of mouse liver DNA (Fig. 3B). Indel analysis at the targeting site by PCR amplification followed by next generation sequencing (NGS) showed various indel mutations in liver DNA from the virus injected mice (Fig. 3C). The indel rate, measured by droplet digital PCR (ddPCR, Fig. 3D), was approximately 2.4% in the mouse treated with a viral dose of 1.2 × 10^12^ vg/kg, compared with 0.05% in the vehicle treated animal. Approximately 0.2%–0.3% mouse liver DNA from the viral vector-treated mice had the *BDD-F8* transgene successfully inserted at the *Alb* locus (Fig. 3D, KI%).

RT-PCR analysis of liver cDNA from the vector-injected mice, at 4 weeks after injection (Fig. 3E), showed both 5′ and 3′ junction fragments at the targeting site. Sanger sequencing (data not shown) confirmed that these fragments contained the correct 5′- and 3′-junction sequences of *Alb-2A-BDD-F8* fusion mRNA, indicating that *BDD-F8* was precisely inserted at the targeted *Alb* locus after CRISPR/Cas9 cleavage. Consistent with these findings, the fusion mRNA was detected in liver from virus-treated, but not vehicle-treated, mice. In contrast, endogenous mouse *Alb* and *F8* mRNA level were not altered (Fig. 3F). Immunofluorescence (Fig. 3G), using specific anti-human FVIII antibody, detected sporadic expression of FVIII protein in the liver cells only in mice treated with viral vectors, not in vehicle controls. Taken together, these results showed that AAV-delivered CRISPR/SaCas9-mediated targeted genome editing of *BDD-F8* transgene into the mouse *Alb* locus led to human BDD-FVIII protein expression in mouse hepatocytes.

### CRISPR/Cas9-NHEJ-mediated genome editing, via AAV delivery, ameliorated hemophilia A phenotype in mice

To evaluate the therapeutic effects of our genome editing approach in a disease state, we generated a mouse model of hemophilia A by deletion of the mouse *F8* gene, abbreviated as F8KO. The C57BL/6 mouse embryos were microinjected with Cas9 protein and two specific gRNAs targeting exon 4 of the mouse *F8* gene, resulting in a 37-bp deletion (Suppl. Fig. 2A,B). WT mice have FVIII activity approximating 30% of human FVIII activity in pooled normal human plasma, based on Chromogenix FVIII activity assays. In comparison, F8KO mice had undetectable plasma FVIII activity (Suppl. Fig. 2C) and exhibited no coagulation, based on an activated partial thromboplastin time (aPTT, Suppl. Fig. 2D). The level of mouse FVIII antigen in these F8KO mice was not measured.

**Figure 4:**
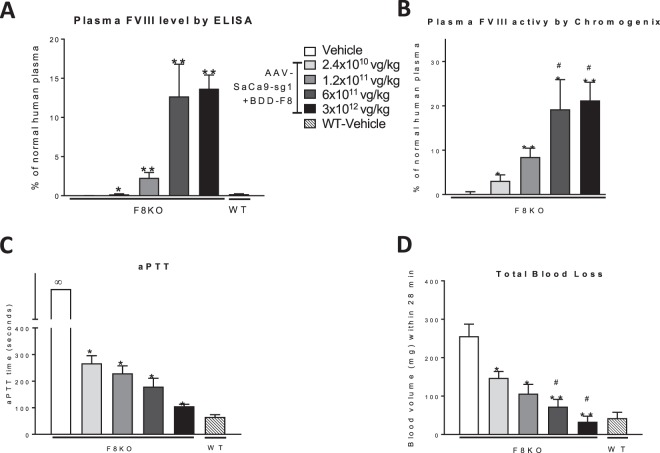
AAV dose-dependent expression of the targeted human *BDD-F8* transgene ameliorated hemophilia A in mice. Adult F8KO or littermate WT control mice were injected without (Veh) or with the indicated total AAV doses of AAV8-SaCas9-sg1 and AAV8-BDD-F8 vectors, mixed at a 1:5 ratio. After 4 weeks, mouse plasma was collected and analyzed for FVIII protein level **(A)**, FVIII activity **(B)** and activated partial thromboplastin time **(**aPTT, **C**). These parameters were assessed by FVIII ELISA, Chromogenix activity assay, and microplate aPTT assay, respectively, as described in Methods. (**D)** Tail vein bleeding time assays performed using filter paper, as described in Methods. Blood was collected into one spot of the filter paper every 4 min, with a total time of 28 min. Blood volume was calculated by a pre-defined standard curve of blood volume vs. spot area. Data are means ± SE. *P < 0.05, **P < 0.01 vs. F8KO mice injected with vehicle; #*P* > 0.3 vs. WT mice injected with vehicle; n = 3–5 mice per group. No coagulation (unlimited aPTT) is indicated by ∞.

These hemophilic F8KO mice received, via a single tail vein injection, AAV8-SaCas9-sg1 and AAV-BDD-F8 vectors, mixed at a 1:5 ratio, at a total dose of 2.4 × 10^10^, 1.2 × 10^11^, 6 × 10^11^, or 3 × 10^12^ vg/kg (Fig. 4). The 1:5 ratio of AAV8-SaCas9-sg1:AAV8-BDD-F8 was selected because it produced the highest hybrid *Alb-2A-BDD-F8* mRNA and FVIII activity levels in F8KO mice, compared with 1:2.5 and 1:20 ratios, all administered at the same total AAV dose (Suppl. Fig. 3). At 4 weeks after injection, F8KO mice receiving viral vectors at all AAV doses had increased plasma human FVIII protein levels, measured by a FVIII ELISA (Fig. 4A). The increase in FVIII levels was dose-dependent and probably reflected only the integrated *BDD-F8* transgene, because the ELISA assay was specific for human, not mouse, FVIII. At a total AAV dose of 6 × 10^11^ or 3 × 10^12^ vg/kg, the plasma FVIII in treated mice reached a FVIII level equivalent to ~13% that in pooled normal human plasma. This peak level of FVIII may be underestimated because FVIII antigen levels were calculated using normal human plasma that contains full length FVIII as a standard; and the full length FVIII and BDD-FVIII proteins may have different binding affinities to the antibodies used in the ELISA assay. Plasma FVIII activity, measured by the chromogenic FVIII activity assay, was also elevated in the vector-treated F8KO mice, in a similar dose-dependent manner (Fig. 4B). Consistent with these findings, aPTT was significantly lower in vector-treated than in vehicle-treated F8KO mice (with the latter, again, showing no clotting during the assay), at all AAV doses, with an apparent dose-dependence (Fig. 4C). A tail bleeding time assay, detecting blood loss volume over 28 minutes further corroborated these results, showing significantly lower blood losses in F8KO mice after treatment of AAV viral vectors (Fig. 4D). In particular, the F8KO mice receiving the highest AAV dose exhibited minimal blood loss. This indicated that the bleeding disorder in the hemophilia A mouse model was ameliorated by the treatment.

### Sustained effects of CRISPR/Cas9-mediated BDD-F8 genome editing in the hemophilia A mouse model

**Figure 5:**
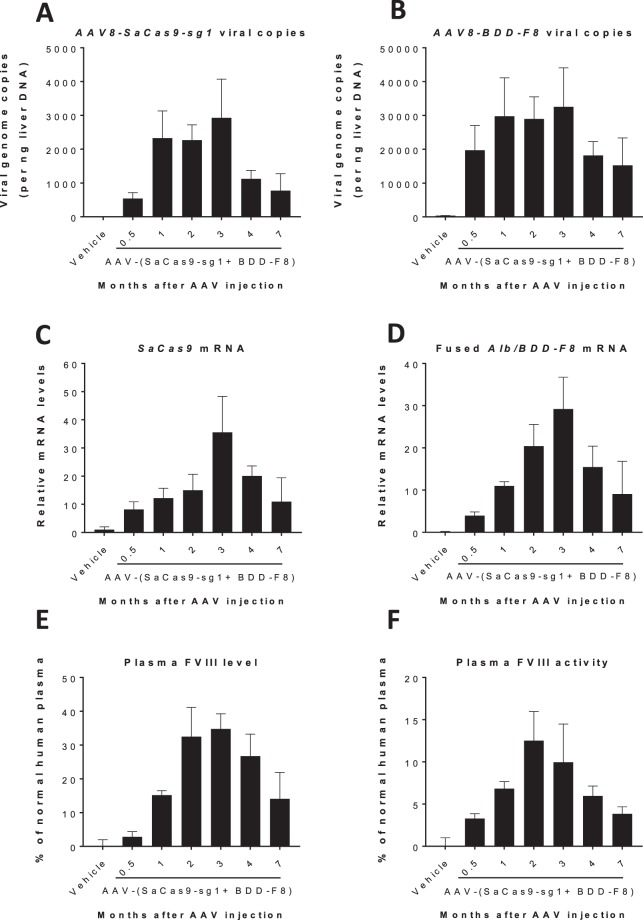
Time course of targeted human *BDD-F8* transgene expression and FVIII activity. F8KO mice were injected without (Veh) or with AAV8-SaCas9-sg1 plus AAV8-BDD-F8 vectors, at a 1:5 ratio and a total AAV dose of 6 × 10^11^ vg/kg. Liver tissue was harvested and plasma collected from mice at the indicated time points after injection. **(A,B)** Viral infectivity in the liver, as indicated by AAV8-SaCas9-sg1 **(A)** and AAV8-BDD-F8 **(B)** viral genome copies, measured by real-time quantitative PCR. **(C,D)** Relative mRNA levels of *SaCas9* **(C)** and hybrid *Alb/BDD-F8* **(D)** in liver cells, measured by real-time RT-PCR. Plasma FVIII protein level **(D)** and activity **(E)**, assessed by FVIII ELISA and Chromogenix activity assay, respectively. Data are means ± SE; n = 3–5 mice in all groups except for the Vehicle group, which had n = 12. In this control group, data were pooled from all time points (0.5–7 months) in all vehicle-injected F8KO mice.

To examine the long-term therapeutic effects of genome editing, adult F8KO mice were treated with either vehicle or the two viral vectors at a 1:5 ratio and a total AAV dose of 6 × 10^11^ vg/kg. Mouse liver and plasma samples were collected at various time points from 0.5 to 7 months (0.5, 1, 2, 3, 4, and 7 months). Copies of both viral genomes in liver cells, detected by real-time quantitative PCR, were evident at all time points, although levels appeared to decrease at 4 months post virus injection (Fig. 5A,B). SaCas9 mRNA was expressed in hepatocytes throughout the entire 7 months, with peak expression at 3 months (Fig. 5C). Unlike the vehicle-treated cohort, that had no detectable fusion mRNA, AAV vector-treated F8KO mice had remarkably elevated levels of *Alb-2A-BDD-F8* fusion mRNA in the hepatocytes (Fig. 5D). In accordance with these changes, plasma FVIII protein (by ELISA detecting FVIII antigen) and procoagulant activity reached ~34% and ~13% of the normal human plasma, respectively (Fig. 5E,F). Notably, at 7 months after AAV injection, the plasma FVIII protein level remained >14% of that in human plasma. Hence, CRISPR/Cas9 mediated genome editing with the human *BDD-F8* transgene maintained its therapeutic function for at least 7 months in mice.

### No indications of liver toxicity or off-target effects in AAV treated F8KO mice

The F8KO mice treated with AAV vectors or vehicle for 0.5–7 months to assess therapeutic persistence (Fig. 5) were also evaluated for signs of liver toxicity. At all time points, there were no difference in liver function or toxicity indicators, including plasma albumin, alanine aminotransferase (ALT), and aspartate aminotransferase (AST) levels, between vehicle- and vector-treated mice (Suppl. Fig. 5A–C). At 2 months, liver histology showed no gross morphological abnormalities in either treatment group (Suppl. Fig. 5D). Mild lymphocyte infiltration around hepatic portal veins was observed in the vector-treated mice (Suppl. Fig. 5E).

To evaluate potential off-target events caused by CRISPR/Cas9, an NGS study was conducted on liver DNA harvested from F8KO mice at 3 months after treatment with vehicle or AAV viral vectors. On-target at the *Alb* locus and 8 potential off-target sites in the mouse genome with up to 3 mismatches, 1 bp insertion or 1 bp deletion, as predicted by the COSMID online program^34^ were examined (Table 1). Samples from vehicle-treated mice yielded 0% indels at all sites. In contrast, in samples from AAV-treated mice, the on-target indel percentage reached 9.38% at the *Alb* locus, whereas off-target indels were undetectable for 6 out of the 8 predicted off-target sites. One off-target site showed 0.58% indel efficiency in an intergenic region with no known gene at the locus and another off-target site showed 1.84% indel efficiency in an intronic region of the *Pard 3* gene. Thus, though further analysis is still required, this evidence indicated that our *in vivo* genome editing approach would be unlikely to produce meaningful off-target cleavage event in the mouse genome.

**Table 1:** On-target and off-target analyses by next generation sequencing (NGS).

On-Target/ Off-Target	gRNA motif (mismatches in bold font)	PAM (NNGRRT)	Chromosome Position	Strand	Gene	Site position	Indel %	
							Vehicle (n = 3)	AAVs (n = 5)
On-target	GTTAGAAATACTTGCAAAGCC	TAGAAT	**Chr5:90474937–90474963**	+	*Alb*	**intronic**	0	**9.38 ± 0.90**
Off-target	GTT**T**GAAATACTT**TT**AAAGCC	GTGAGT	Chr5:3757359-3757385	−	*Ankib1*	intronic	0	0
Off-target	**T**TTAGAAATACTT**AA**AAAGCC	AAGAGT	Chr8:77780734-77780759	+	*NA*	intergenic	0	0.58 ± 0.58
Off-target	G**A**TAGAAA**C**ACTTGCAAA-CC	ATGAAT	Chr8:127199445-127199470	−	*Pard3*	intronic	0	1.84 ± 0.76
Off-target	GTTA**A**GA-**C**ACTTGCAAAGCC	AAGGAT	Chr11:54929261-54929286	+	*Tnip1*	intronic	0	0
Off-target	GTTAGAAATAC**C**TGCAAAGC-	TAAGGT	Chr12:7607521-7607546	+	*NA*	intergenic	0	0
Off-target	GTTA**A**AAA**C**ACTTGCA**G**AAGCC	CTGGGT	Chr12:111780123-111780150	−	*Klc1*	intronic	0	0
Off-target	**T**TTAGAAATACT**A**GC-AAGCC	TGGGAT	Chr19:28248970-28248995	−	*NA*	intergenic	0	0
Off-target	GTTAGAAAT**T**CTTG**G**AA**T**GCC	TAGGGT	ChrX:15412386-15412412	+	*NA*	intergenic	0	0

F8KO mice were injected without (Vehicle, n = 3) or with AAV8-SaCas9-sg1 plus AAV8-BDD-F8 viruses, at a 1:5 ratio and a total AAV dose of 6 × 10^11^ vg/kg (n = 5). After 3 months, mice were euthanized and their liver DNA was purified for an NGS study. On-target at the *Alb* locus and 8 potential off-target sites with up to 3 mismatches, 1 bp-insertion or deletion, as predicted by the COSMID online program (http://crispr.bme.gatech.edu), were evaluated. NA, no gene available at this locus.

## Discussion

This study demonstrated that *in vivo* genome targeting of the human *BDD-F8* transgene into the *Alb* locus by CRISPR/Cas9, leading to human FVIII production in the liver, ameliorated severe hemophilia A phenotype in mice. Furthermore, the beneficial effects lasted for at least 7 months, with no indication of liver toxicity and no meaningful off-target effects, supporting the hypothesis that site-specific genome editing would enable permanent FVIII replacement. This is an improvement not only to current treatments involving parenteral administration of FVIII preparations, but also to AAV mediated gene therapy approaches, which deliver vectors maintained as episomes. Thus, these findings support the potential promise of an analogous genome editing approach, using CRISPR/Cas9 and the appropriate AAV vectors, for FVIII replacement in patients with hemophilia A.

Due to the large size of *BDD-F8*, we developed an approach using CRISPR/Cas9 with, instead of the traditional HDR, NHEJ-mediated site-specific insertion. This not only made gene editing with *BDD-F8* feasible, but also resulted in a higher knock-in efficiency than with HDR. Using AAV delivery in HEK-293 cells, we found that the CRISPR/Cas9 NHEJ-mediated gene knock-in efficiency was from 4- to over 6-fold higher than the CRISPR/Cas9 HDR pathway at the *GAPDH* locus. The CRISPR/Cas9 NHEJ approach not only made it feasible to package a large gene such as *BDD-F8* in AAV, but it also offered the potential for higher knock-in efficiency. In our proof-of-concept mouse study, indeed, this approach resulted in therapeutic levels of BDD-FVIII in the plasma and ameliorated the hemophilia A phenotype.

Our approach inserted *BDD-F8* at a new location, the intron 13 region of the *Alb* locus, leading to production of a polycistronic transcript of albumin-2A-BDD-F8 mRNA, under control of the endogenous *Alb* promoter. This mRNA was then translated into two functional proteins, albumin and BDD-FVIII. On the other hand, if the transgene cassette is inserted in an opposite orientation, no *BDD-F8* would be expressed. Despite placing human *BDD-F8* expression under control of the *Alb* promoter, our genome editing method did not impact normal albumin production in the mice, as evidenced by levels of albumin mRNA (Fig. 3G) and protein (Suppl. Fig. 5A) in vector- compared with vehicle-treated mice. By using the *Alb* promoter, this approach ensured liver-specific expression of BDD-FVIII because, in normal human plasma, albumin levels are about one million times higher than those of FVIII. Even considering other factors that would impact FVIII levels, such as protein folding, secretion, and transport across cell membranes and the fact that FVIII is normally expressed in liver sinusoidal endothelial cells, BDD-FVIII expression under the *Alb* promoter should still be higher than under its own *F8* promoter. As a result, a BDD-F8 knock-in event in even a very small fraction of hepatocytes would potentially produce therapeutically active levels of BDD-FVIII. This was, indeed, what we observed in the treated F8KO mice. Our ddPCR experiments showed an approximately 0.2%–0.3% BDD-F8 knock-in efficiency after 4 weeks (Fig. 3D). This corresponded to 7% of the normal human plasma activity levels of BDD-FVIII, and, after 2 months, a further increase to ~13%, above that required in patients for therapeutic activity.

In the time course study, administering a total dose of 6 × 10^11^/kg AAV8-(SaCas9-sg1 + BDD-F8), saCas9 and BDD-F8 mRNAs and BDD-FVIII protein and activity were first detectable at 2 weeks after vector injection, steadily increasing to approximately 13% procoagulant activity at 2 months (Fig. 5). This time lag may reflect the need for the viral DNA to be in a double-stranded form for *BDD-F8* transgene insertion into the genome. The process of converting single-stranded to double-stranded DNA in the cells may take some time. A gradual decrease in viral copy numbers and mRNA and protein levels was observed at 4–7 months after viral vector injection. The reason for this decrease needs to be further investigated. There are several possible causes for this decrease. Since the study was done in immunocompetent mice, they are likely to develop anti-human FVIII inhibitors and AAV vectors could be diluted and eliminated over time due to immune response. Other possible explanations could be cell death and elimination of genetically modified cells due to genomic instability, apoptosis, or anti-Cas9 immune reaction, although no anti-Cas9 immune reaction was detected in non-human primates using the same approach (data not shown).

Hemophilia A is a monogenetic hereditary disorder and is, therefore, a target for gene therapy, especially because its genetics are well understood. Clinical trials using AAV-mediated gene therapy showed both safety and delivery efficiency. In trials by Biomarin, *BDD-F8* delivered using AAV5 resulted in sustained plasma levels of BDD-FVIII, equivalent to >50% of normal FVIII levels, for over 2 years (World Federation of Hemophilia, Glasgow, 2018^35^). At the same conference, trials sponsored by Spark Therapeutics were reported to demonstrate BDD-FVIII levels >10% normal FVIII levels, using a viral dose ~100-fold lower than that used in the Biomarin study. The Spark study used a modified AAV8 serotype for delivery.

However, an AAV-based gene therapy approach still has limitations in that expression of therapeutic genes may not be permanent, as the viral genome containing the therapeutic gene is maintained as an episome. Furthermore, repeat administration is generally prohibitive because of humoral immune responses against AAV capsid proteins, generally developing after the first administration^24^. In contrast, gene editing approaches, demonstrated for FVIII in our study and for Factor IX by Ohmori *et al*.^36^ and Sangamo Therapeutics (using zinc finger nucleases), can potentially offer a life-time cure for hemophilia patients. Most important, early administration of a permanent genome-editing treatment to pediatric patients would spare them from ever needing to undergo coagulation factor infusions that, in current practice, can begin at very young ages^37^. It should also be noted that, in infants, with cell proliferation more rapidly diluting the AAV genome, the likelihood of side effects associated with the CRISPR/Cas nuclease would be lower than in adults. In addition, because infants have a lower frequency of endogenous neutralizing antibodies to AAV serotypes, certain AAV-based treatments might be more efficient in infants than in adults^38^. While our study included only adult F8KO mice, we would expect our strategy to also be applicable to younger subjects.

CRISPR/Cas9 has distinctive advantages over zinc finger nucleases (ZFN) or transcription activator-like endonucleases (TALEN) for treatment of genetic diseases, although all three approaches involve making double stranded DNA breaks. ZFN-mediated gene editing corrected the hemophilia A and B phenotype in mice^39^ and, in that study, FVIII reached about 35% level. Despite the efficiency of gene editing by ZFN, it has not been widely adopted because it is difficult to construct zinc-finger domains binding a comprehensive stretch of nucleotides with sufficiently high specificity and affinity. In contrast to protein-guided DNA cleavage, CRISPR/Cas9 employs a synthetic sgRNA to target a specific DNA locus and is, therefore, flexible and easy to develop. This feature is especially important to enable personalized genome editing, that is, modification of patient-specific gene mutations. Here, for the first time we have shown that genomic-integrated FVIII using CRISPR/Cas9 corrected a hemophilia A mouse phenotype.

At all target patient ages, concerns about use of these technologies, including off-target mutations potentially caused by CRISPR/Cas, AAV insertions possibly causing genotoxicity, and adverse events triggered by host immune responses to the therapy, will necessitate extensive safety evaluations during clinical trials. We observed no evidence of liver toxicity in treated F8KO mice monitored for 7 months after viral vector injection. It was also promising that Ohmori *et al*. did not observe signs of tumor formation in mice up to 12 months after CRISPR/Cas9-mediated gene editing in hemophilia B mice^36^. Off-target events have been a potential safety concern in gene editing-based therapies^40^. Using NGS, we examined top 8 potential off-target sites, predicted using the COSMID online tool. Only one site was detected, with an indel frequency <2%. This site is in an intron region, so is unlikely to cause meaningful safety concerns. This low incidence of off-target events was consistent with reports by Iyer *et al*.^41,42^. Notably, no increased incidence of cancer has been noted, so far, in the several clinical studies of AAV-mediated gene therapies^36^.

## Conclusions

Together, our findings demonstrated that the hemophilia A phenotype in F8KO mice was corrected by site-specific genome editing to drive liver-specific production of an active form of human FVIII. Furthermore, these benefits lasted for at least 7 months and not associated with liver toxicity. Overall, these findings support development of a comparable genome editing approach for achieving permanent FVIII replacement in patients with hemophilia A.

## Supplementary information

Hemophilia A ameliorated in mice by CRISPR-based in vivo genome editing of human Factor VIII

## Data Availability

The datasets generated during and/or analyzed during the current study are available from the corresponding author on reasonable request. Raw sequence data of DNA fragments/constructs are deposited in GenBank with the following accession codes: BankIt2270460 SaCas9 MN548085; BankIt2270466 pCDNA4-BDD-F8 MN548086; BankIt2270469 px602-BDD-F8 MN548087.
