# Supplementary material for: Hemophilia A ameliorated in mice by CRISPR-based in vivo genome editing of human Factor VIII
Source: Sci Rep. 2019 Nov 14;9:16838. doi: 10.1038/s41598-019-53198-y (PMC6856096; doi:10.1038/s41598-019-53198-y)

Hemophilia A ameliorated in mice by CRISPR-based *in vivo* genome editing of human Factor VIII

Hainan Chen, Mi Shi, Avital Gilam, Qi Zheng, Yin Zhang, Ivka Afrikanova, Jinling Li, Zoya Gluzman, Ruhong Jiang, Ling-Jie Kong*, Ruby Yanru Chen-Tsai*

*Corresponding authors: Ruby Yanru Chen-Tsai and Ling-Jie Kong

Applied Stemcell, Inc., 521 Cottonwood Drive, Milpitas, CA 95035

Supplementary Table 1. Probes for ddPCR assays for BDD-FVIII and SaCas9 viral titers.

Assay Target	Product size	Primer / Probe	Sequence	Length (bp)	Tm, °C	%GC	Vendor
BDD-F8	121bp	Primer Fwd	5'-/ ACAGTTCAGGGATCAAGCACAAC / -3'	23	62.7	47.8	IDT
		Primer Rev	5'-/ AGATCGCACCCCATCAGTTC / -3'	20	62.4	55	
		Probe	5'-/ 56-FAM/TAGATACAT/ZEN/CAGGCTGCACCCAACCCAT/3IABkFQ/ -3'	28	71.5	50	
SaCas9	103bp	Primer Fwd	5'-/ AACGAAGAGGATATTAAGGGCTACAG / -3'	26	61.4	42.3	IDT
		Primer Rev	5'-/ CTTTCCGGGCGGTAATGTC / -3'	19	62.7	57.9	
		Probe	5'-/ 56-FAM/ACCGGCAAG/ZEN/CCCGAGTTCACC/3IABkFQ/ -3'	21	71.7	66.7	

Supplementary Table 2. Custom-designed PCR primers used in this study

Gene/Virus	position	Forward (5'-3')	Reverse (5'-3')	Purpose	Assays
Alb	intron 13	ACGTTTTTGCATTTTGACGA	GTAAACGTACGTGCCTTGCAT	gRNA cleavage efficiency	T7E1 surveyor assay, Sanger sequencing
Alb	exon 4	TTCCAAACCTCCGTGAAAAC	AAGGTGGTTGGGTTTTCCTT	mRNA level	RT-PCR, SYRB green quantitative RT-PCR
cyclophilin A	exon 1 & 3	CACCGTGTTCTTCGACATCA	TGGCACATGAATCCTGGAA	mRNA level	SYRB green quantitative RT-PCR
F8	exon 2 & 3	CAAGGACCAGCTTTTCAACA	AGGACACACCAACAGCATGA	mRNA level	SYRB green quantitative RT-PCR
F8	exon 4	GATCAGACAAGCCAAATGGAGAAG	GCCCTTTTCAGAACAAAGAGTTC	*F8KO* mouse genotyping	PCR
(human WT) F8	exon 1 & 2	GTGCAGTGGAACTGTCATGG	CGACTGAGGTGTTGAATGGA	mRNA level	SYRB green quantitative RT-PCR
Fused *Alb-BDD-F8*	5'-junction	TACAGCGGAGCAACTGAAGA	TAGACCACGCTGGTGTTGAA	Fused *Alb-BDD-F8* mRNA level	RT-PCR, SYRB green quantitative RT-PCR
Fused *Alb-BDD-F8*	3'-junction	AGTCCTGGGTGCATCAGATT	GCCTGAGAAGGTTGTGGTTG	Fused *Alb-BDD-F8* mRNA level	RT-PCR, SYRB green quantitative RT-PCR
AAV-*BDD-F8*		ACGTGAGCAACAACAGCAAC	TAATGCCGGGTTTTCTTCTG	Virus infection rate, *BDD-F8* mRNA level	PCR, SYRB green quantitative PCR/RT-PCR
AAV-*SaCas9-sg1*		GAGTGGCCAACTCCATCAC	ATACCGACCTTCCGCTTCTT	Virus infection rate	PCR, SYRB green quantitative PCR
AAV-*SaCas9-sg1*		CGCGAGTACCTGGAAAACAT	CCGCTAGCGTAATCTGGAAC	*SaCas9* mRNA level	SYRB green quantitative RT-PCR

Supplementary Table 3. Custom-designed PCR primers used for on-target and off-target analyses by NGS

Chromosome Position	Gene	Forward (5'-3')	Reverse (5'-3')
Chr5:90474937-90474963	*Alb*	**TCGTCGGCAGCGTCAGATGTGTATAAGAGACAG**TGCCACACTGCTGCCTATTA	**GTCTCGTGGGCTCGGAGATGTGTATAAGAGACAG**TGTTGTGCCCAAAATAAGAAGA
Chr5:3757359-3757385	*Ankib1*	**TCGTCGGCAGCGTCAGATGTGTATAAGAGACAG**TCAGCTCGTTTGTCATCAGG	**GTCTCGTGGGCTCGGAGATGTGTATAAGAGACAG**TGGTCAGGTCCAGAAAACTG
Chr8:77780734-77780759	*NA*	**TCGTCGGCAGCGTCAGATGTGTATAAGAGACAG**TTGCACTGTGCTCATGGACT	**GTCTCGTGGGCTCGGAGATGTGTATAAGAGACAG**TTTGCCTGATCTGTCAATGC
Chr8:127199445-127199470	*Pard3*	**TCGTCGGCAGCGTCAGATGTGTATAAGAGACAG**TGGATCTCATAGAGGCGTGA	**GTCTCGTGGGCTCGGAGATGTGTATAAGAGACAG**TGGCAGAGACTGAAGGTGTG
Chr11:54929261-54929286	*Tnip1*	**TCGTCGGCAGCGTCAGATGTGTATAAGAGACAG**CTTTGCAGCTGCTGTTCATC	**GTCTCGTGGGCTCGGAGATGTGTATAAGAGACAG**CAACCTACAGATGGGGGAAA
Chr12:7607521-7607546	*NA*	**TCGTCGGCAGCGTCAGATGTGTATAAGAGACAG**GCTATGATACCCTTGCAGTAGGA	**GTCTCGTGGGCTCGGAGATGTGTATAAGAGACAG**GCCTCCAGGGAGATTTTTGT
Chr12:111780123-111780150	*Klc1*	**TCGTCGGCAGCGTCAGATGTGTATAAGAGACAG**AGCTGAAGCTGCAGATGGTT	**GTCTCGTGGGCTCGGAGATGTGTATAAGAGACAG**CACCCTGGTCCCTCTTTACA
Chr19:28248970-28248995	*NA*	**TCGTCGGCAGCGTCAGATGTGTATAAGAGACAG**TTGACTTGAGCATCTGTACCAA	**GTCTCGTGGGCTCGGAGATGTGTATAAGAGACAG**TCATAAGTGTGCATAGCTTGCTT
ChrX:15412386-15412412	*NA*	**TCGTCGGCAGCGTCAGATGTGTATAAGAGACAG**CCCAGCTCTGGTGTTTTAGC	**GTCTCGTGGGCTCGGAGATGTGTATAAGAGACAG**GTTCCTGATAGCCCCACAAA

For library preparation and NGS sequencing, Illumina Nextera adaptor (Bold font) was added to each locus-specific sequence (regular font).

Supplementary Table 4. Custom designed ddPCR assays to detect Indel and F8 knock-in in the study

Assay name	Purpose	Vendor	Product size	Primer/probe name	Sequence	Position
Alb indel assay	Indel detection	Bio-Rad	Bio-Rad proprietary info	Forward (5'-3')	Bio-Rad proprietary info	Alb
				Reverse (5'-3')	Bio-Rad proprietary info	Alb
				5' FAM/ZEN/3' IB®FQ Probe (indel)	Bio-Rad proprietary info	Alb intron 13, on cut site
				5' HEX/ZEN/3' IB®FQ Probe (reference)	Bio-Rad proprietary info	Alb
BDD-F8_KI assay	BDD-F8 KI detection	IDT	180bp	Forward (5'-3')	CACCTGTGGTCAACTCTCTG	BDD-F8
				Reverse (5'-3')	GAAACATTTCAGGGCAAGGT	Alb intron 13
				5' FAM/ZEN/3' IB®FQ Probe	TGCAATCTGATGCACCCAGG	BDD-F8
Reference assay	Reference region	IDT	220bp	Forward (5'-3')	GCAAGTTCTTAGTTGGCACC	Chr3:18144568-18144913
				Reverse (5'-3')	ATGAGCATGCAACACTCTGT	Chr3:18144568-18144913
				5' HEX/ZEN/3' IB®FQ Probe	TCCTCTCTCGCACAGCATCT	Chr3:18144568-18144913

Supplementary Figure legends

Supplementary Figure 1. Comparison of NHEJ- and HDR-mediated genome editing efficiencies by CRISPR/Cas9 in HEK-293 cell lines stably expressing SpCas9. (A) Schematic diagrams showing knock-in strategy at the *GAPDH* locus. As illustrated, HEK-293 cells stably expressing SpCas9 were co-transduced with AAV2-U6-sgRNA targeting the *GAPDH* locus and AAV2-donor viruses expressing an IRES-controlled GFP transgene. Three AAV2-donor vectors were tested, comprised of long (~900 bp), or short (~150 bp) homologous arms, or only gRNA-PAM targeting sequences (~27 bp), as indicated. If cleaved by SpCas9/gRNA, HDR/knock-in will occur with the first two vectors and NHEJ/knock-in with the third. **(B)** Representative live cell images showing that, after AAV2 transduction for 2 weeks, there were many more GFP^+ cells (green) in cultures transduced by the AAV2 donor vector with a gRNA-PAM arm. **(C)** FACS analyses of GFP^+ HEK-293 cells after transduction with the vectors, as indicated. GFP^+ cell population ratios were 0.19% (Control), 1.52% (long homologous arm), 0.92% (short homologous arm), and 6.05% (gRNA-PAM arm) in cells co-transduced with the AAV2-sgRNA vector.

Supplementary Figure 2. Characterization of F8KO mice. (A) F8KO mice were generated in the C57BL/6 strain by targeting the mouse *F8* exon 4, using CRISPR/Cas9 and two specific sgRNAs, as shown. A 37-bp deletion was confirmed by Sanger sequencing at the *F8* exon 4 locus. **(B)** Representative genotyping results from 4 F8KO offspring. The male mice were either hemizygotes with only the KO allele (265 bp) or WT with only the WT allele (302 bp). In contrast, the female mice were either homozygous KO or heterozygotes, containing both KO and WT alleles.

(C) Plasma FVIII activity in male WT and F8KO mice, measured by the Chromogenix FVIII activity assay. **P* <0.01, n = 4 for WT and n = 3 for F8KO. **(D)** Microplate-based aPTT assay results from F8KO and WT mouse plasma samples. In this assay, once a coagulation reaction occurs, absorbance at 405 nm dynamically increases. The aPTT is defined as the time point at which the coagulation reaction reaches its maximum velocity. Unlike that from WT mice, plasma from F8KO mice never coagulated. Data are means ± SE for n = 3–5 mice per group.

Supplementary Figure 3. *In vivo* genome editing efficiency with various ratios of AAV8-SaCas9-sg1 and AAV8-BDD-F8. Adult F8KO mice were injected without virus (Vehicle) or with AAV8-SaCas9-sg1 and AAV8-BDD-F8 viruses at a total AAV dose of $3x10^{12}$ vg/kg, but at various ratios, 1:2.5, 1:5 or 1:20. After 4 weeks, livers were harvested for total RNA purification and plasma was collected, and these samples were analyzed, as indicated. **(A)** Schematic diagrams showing mouse *Alb* and fused *Alb/BDD-F8* mRNAs. **(B)** Ratios of fused *Alb/BDD-F8* mRNA over *Alb* mRNA, measured by SYBR green real-time RT-PCR using primer sets indicated in **A** (red arrows). **(C)** Plasma FVIII activity, assessed by the Chromogenix activity assay. Data are means ± SE for n = 2 mice per group. **(D)** Representative photos of mice undergoing tail bleeding time assays, taken at the end of the experiment (28 minutes). As magnified in the circles, substantially smaller blood drops (indicating an improved blood clotting process) were hanging over the tail tips of the F8KO mice injected with the two AAV viruses at a ratio of 1:2.5 or 1:5, compared with those of mice injected without virus (Vehicle) or with the AAV viral vectors at a 1:20 ratio.

Supplementary Figure 4. Liver function and toxicity assessments in F8KO mice after AAV vector injection. F8KO mice were injected without virus (Veh) or with AAV8-SaCas9-sg1 plus AAV8-BDD-F8 vectors, at a 1:5 ratio and total AAV dose of $6x10^{11}$vg/kg. **(A–C)** Liver panel testing results for plasma albumin, ALT, and AST levels. Data are means ± SE for n = 3–5 mice per group. The values obtained in these assays were indistinguishable between AAV- and vehicle-treated animals at all time points. **(D–E)** Hemotoxylin and eosin stained liver sections, obtained from F8KO mice at 2 months after AAV or vehicle injection. Histological analysis indicated no remarkable gross morphological changes in the livers, other than a mild degree of lymphocyte infiltration around hepatic portal veins (**E**). Scale bars, 10 μm.

Supplementary Figure 5. Full-length gel images of the cropped gels shown in Figure 2C.

Supplementary Figure 6. Full-length gel images of the cropped gels shown in Figure 3. (A) Full length gel image for cropped gels in Figure 3B. The right side of the gel (labeled in yellow) are PCR fragments amplified by BDD-F8 specific primers and was cropped to generate the top gel in Figure 3B. The left side of the gel (labeled In blue) are PCR fragments amplified by saCas9 specific primers and was cropped to generate the bottom gel image in Figure 3B. **(B-D)** Full-length gel for the top, middle and bottom image, respectively in Figure 3E. The red rectangular box indicates the area that was cropped.

Supplementary Figure 1

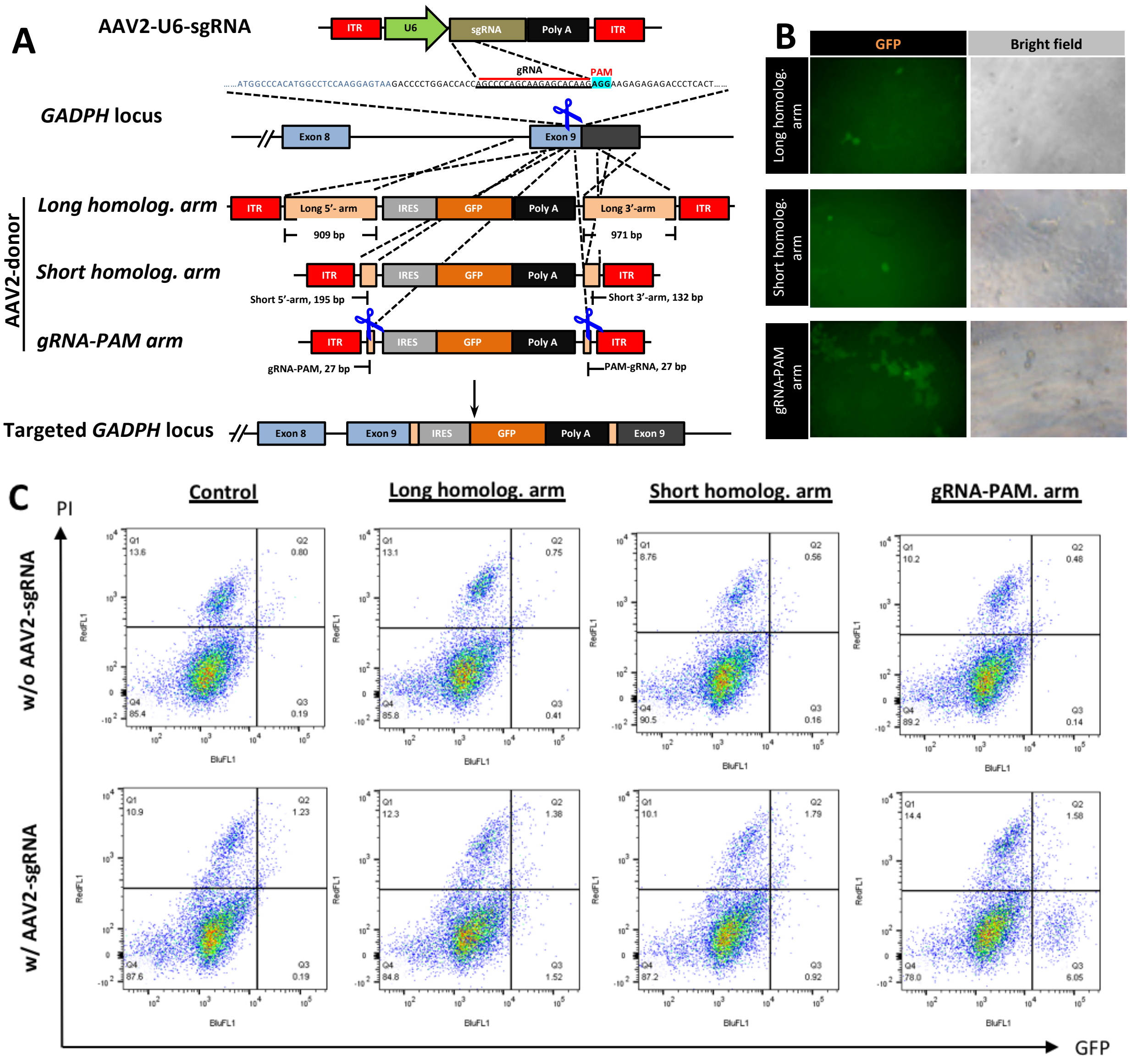

Supplementary Figure 2

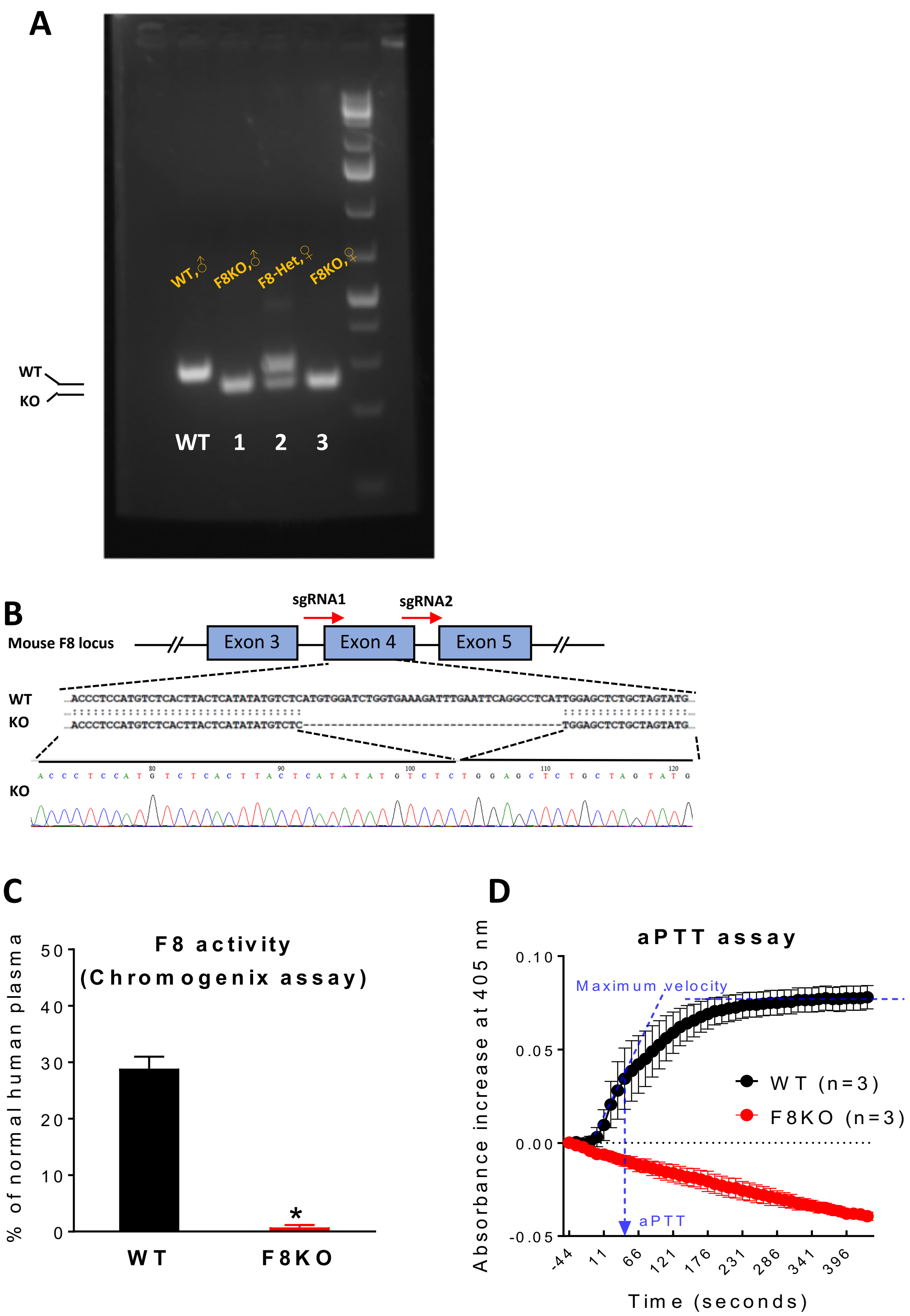

Supplementary Figure 3

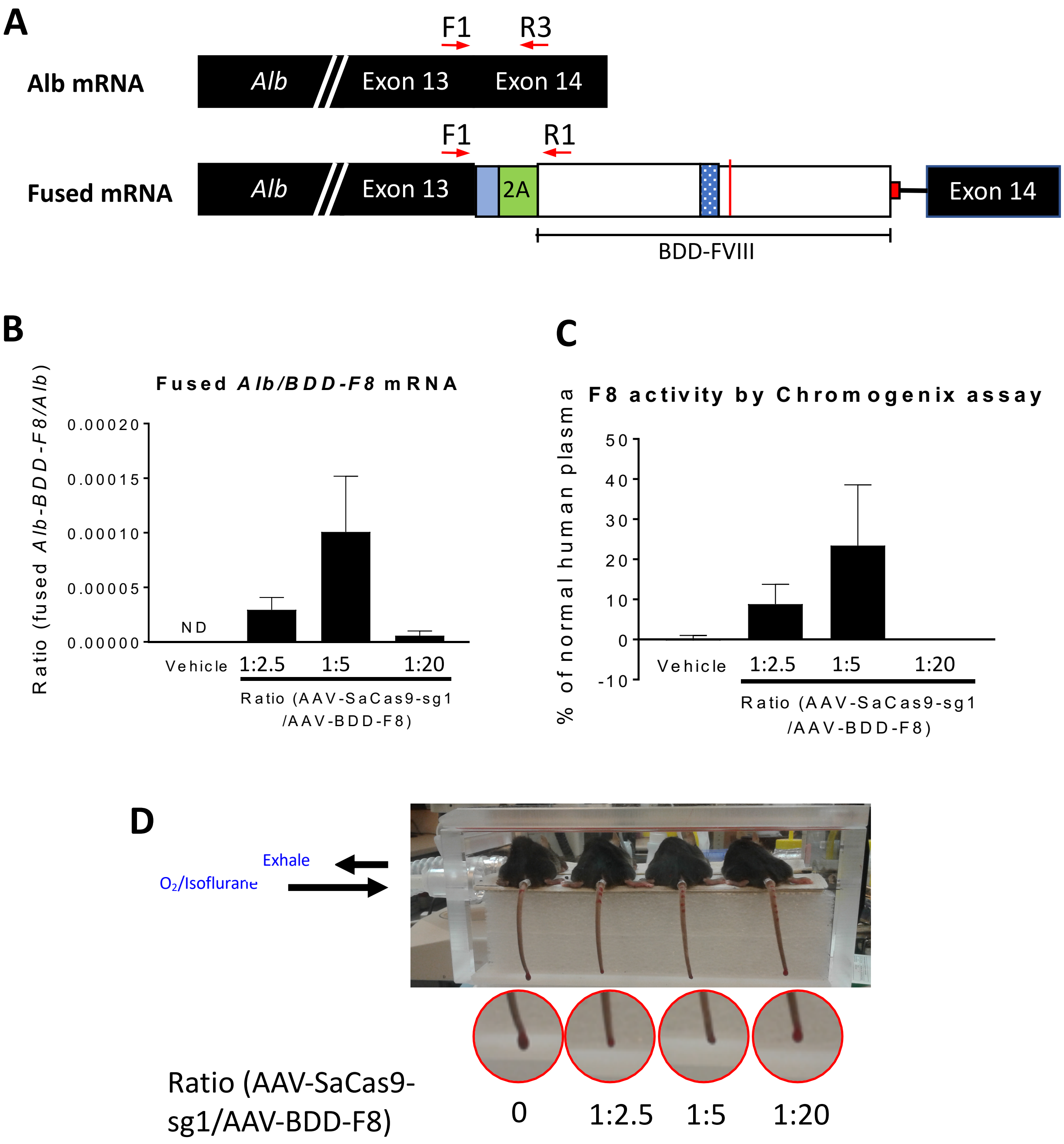

Supplementary Figure 4

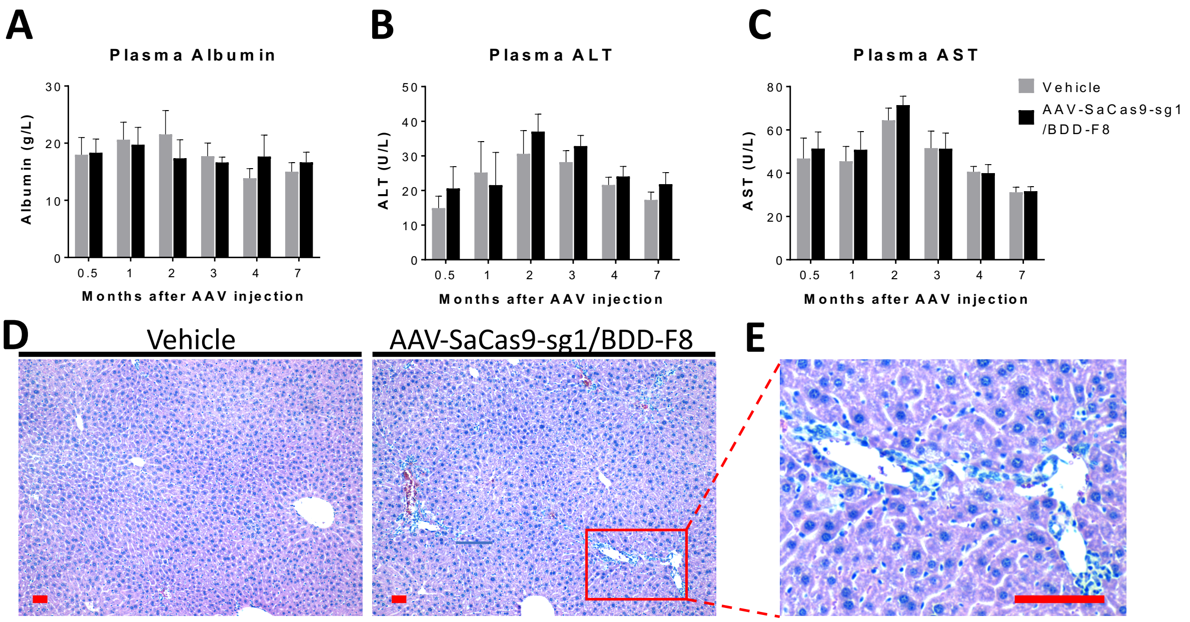

Supplementary Figure 5

C

MEF cells

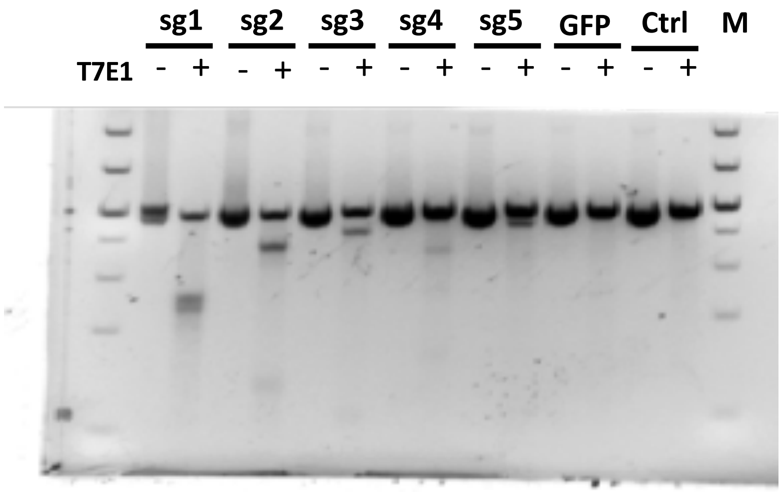

Mut. % 40% 35% 21% 6% 13%

Hepa 1-6 cells

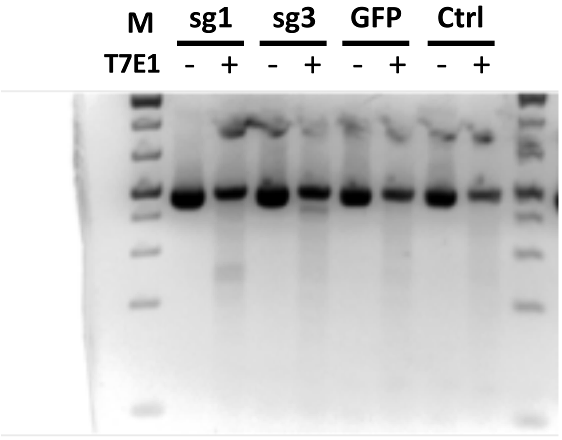

Mut. % 20% 15%

Supplementary Figure 6

A. Full-length gel for Figure 3B

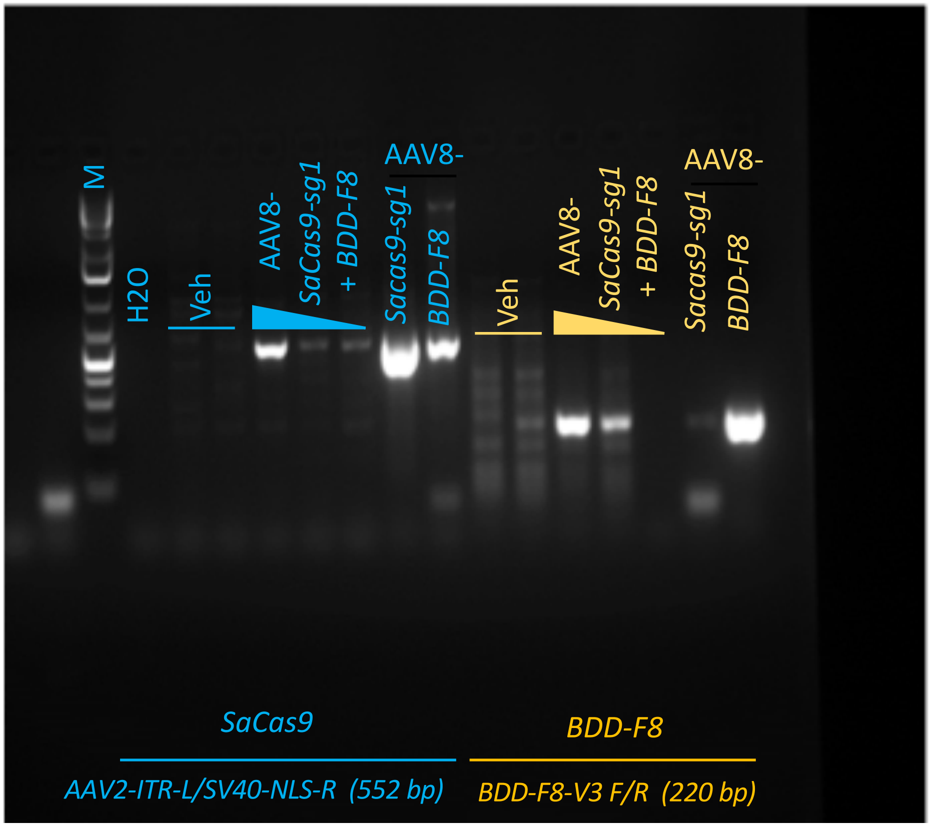

Supplementary Figure 6 (continued)

B. Full-length gel for the top image in Figure 3E

5'-Junction PCR

Expected PCR band at 393kb

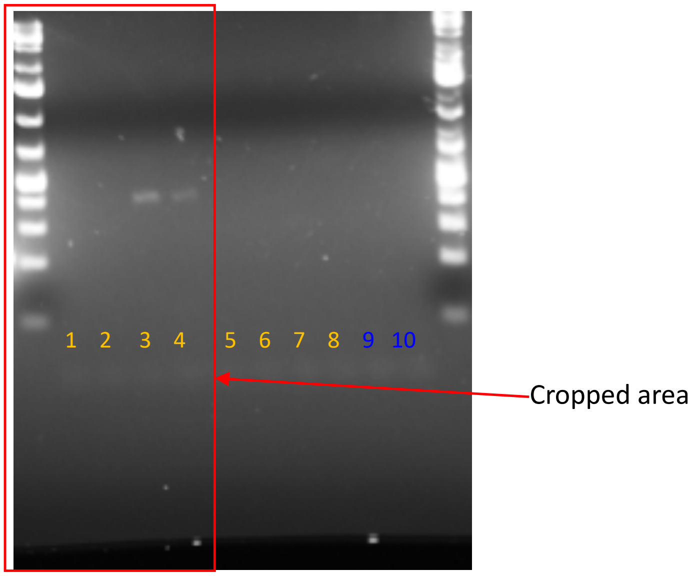

1, 2: Veh
3, 4: High dose AAV8-SaCas9-sg1+BDD-F8
5, 6: middle dose
7, 8: low dose
9: AAV8-BDD-F8 virus
10: AAV8-SaCas9-sg1 virus

C. Full-length gel for the middle image in Figure 3E

3'-Junction PCR

1, 2: Veh
3, 4: AAV8-SaCas9-sg1+BDD-F8 (high dose)

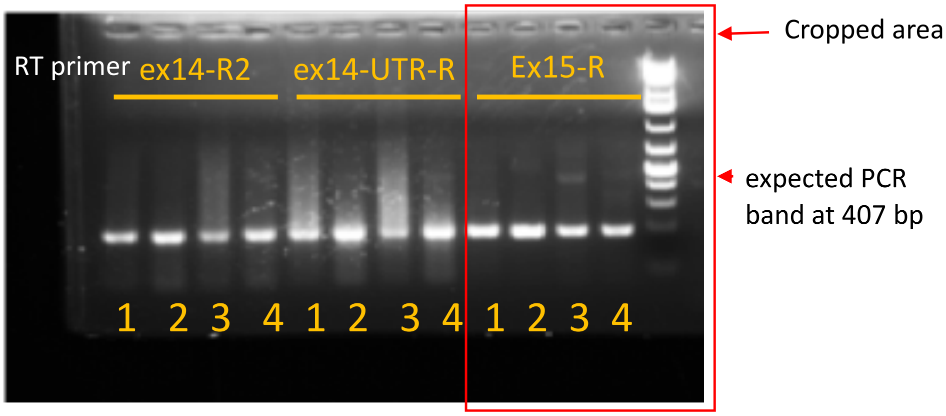

D. Full-length gel for the bottom image in Figure 3E

***Alb* exon 4**

1, 2: Veh
3, 4: High dose AAV8-SaCas9-sg1+BDD-F8
5, 6: middle dose
7, 8: low dose
9: AAV8-BDD-F8 virus
10: AAV8-SaCas9-sg1 virus

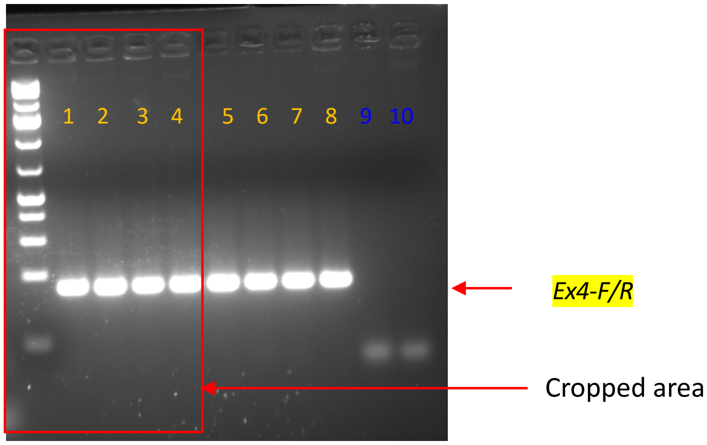

Supplement: Supplementary file 1 — Hemophilia A ameliorated in mice by CRISPR-based in vivo genome editing of human Factor VIII [file 41598_2019_53198_MOESM1_ESM.pdf]
